# Identification of resistant germplasm containing novel resistance genes at or tightly linked to the *Pi2/9* locus conferring broad-spectrum resistance against rice blast

**DOI:** 10.1186/s12284-017-0176-z

**Published:** 2017-08-04

**Authors:** Gui Xiao, Frances Nikki Borja, Ramil Mauleon, Jonas Padilla, Mary Jeanie Telebanco-Yanoria, Jianxia Yang, Guodong Lu, Maribel Dionisio-Sese, Bo Zhou

**Affiliations:** 1 Genetics and Biotechnology Division, International Rice Research Institute, DAPO Box 7777, Metro Manila, Philippines; 2 Institute of Biological Sciences, University of the Philippines Los Baños, 4031 Laguna, Philippines; 3 Fujian Agriculture and Forest University, Fuzhou, 350002 China

**Keywords:** *Magnaporthe oryzae*, *Pi2/9* homologues, Resistant haplotype specific marker

## Abstract

**Background:**

The rice *Pi2/9* locus harbors multiple resistance (*R*) genes each controlling broad-spectrum resistance against diverse isolates of *Magnaporthe oryzae*, a fungal pathogen causing devastating blast disease to rice. Identification of more resistance germplasm containing novel *R* genes at or tightly linked to the *Pi2/9* locus would promote breeding of resistance rice cultivars.

**Results:**

In this study, we aim to identify resistant germplasm containing novel *R* genes at or tightly linked to the *Pi2/9* locus using a molecular marker, designated as Pi2/9-RH (*Pi2/9* resistant haplotype), developed from the 5′ portion of the *Pi2* sequence which was conserved only in the rice lines containing functional *Pi2/9* alleles. DNA analysis using Pi2/9-RH identified 24 positive lines in 55 shortlisted landraces which showed resistance to 4 rice blast isolates. Analysis of partial sequences of the full-length cDNAs of *Pi2/9* homologues resulted in the clustering of these 24 lines into 5 haplotypes each containing different *Pi2/9* homologues which were designated as *Pi2/9-A5*, −*A15*, −*A42*, −*A53*, and -*A54*. Interestingly, *Pi2/9-A5* and *Pi2/9-A54* are identical to *Piz-t* and *Pi2*, respectively. To validate the association of other three novel *Pi2/9* homologues with the blast resistance, monogenic lines at BC_3_F_3_ generation were generated by marker assisted backcrossing (MABC). Resistance assessment of the derived monogenic lines in both the greenhouse and the field hotspot indicated that they all controlled broad-spectrum resistance against rice blast. Moreover, genetic analysis revealed that the blast resistance of these three monogenic lines was co-segregated with Pi2/9-RH, suggesting that the *Pi2/9* locus or tightly linked loci could be responsible for the resistance.

**Conclusion:**

The newly developed marker Pi2/9-RH could be used as a potentially diagnostic marker for the quick identification of resistant donors containing functional *Pi2/9* alleles or unknown linked *R* genes. The three new monogenic lines containing the *Pi2/9* introgression segment could be used as valuable materials for disease assessment and resistance donors in breeding program.

**Electronic supplementary material:**

The online version of this article (doi:10.1186/s12284-017-0176-z) contains supplementary material, which is available to authorized users.

## Background

Rice blast, a devastating rice disease caused by the fungal pathogen *Magnaporthe oryzae,* is affecting rice production across all rice-growing areas worldwide (Ashkani et al. 2014). Introgression of resistance (*R*) genes into rice cultivars remains the most economical and effective approach for rice blast disease management (Ashkani et al. 2015; Tanweer et al. 2015). The bottleneck of this approach is that, after an individual *R* gene is isolated and deployed in the varieties, it can be overcome in a short time (usually in 2–3 years) by the emergence of a compatible pathogen because of the high level of avirulence (*Avr*) effector variability in the pathogen (Skamnioti and Gurr 2009; Valent and Khang, 2010). Therefore, it is essential to enrich and diversify the *R*-gene pool by extensive and continuous exploration of novel *R* genes or alleles in diverse germplasm for the choice of effective *R* genes in a rice resistance breeding program.

To date, more than 100 rice blast *R* genes and over 350 resistance quantitative trait loci (QTLs) have been genetically identified (Tanweer et al. 2015). Of the 100 *R* genes, 25 were molecularly characterized (Liu et al. 2013; Fukuoka et al. 2014; Su et al. 2015; Ma et al. 2015; Chen et al. 2015). Most of them encode proteins having nucleotide binding site (NBS) and leucine-rich repeat (LRR) domains. It is evident that many NBS-LRR-type *R* genes are organized as alleles located at the same loci (Leung et al. 2015). For example, at least eight *Pik* alleles were molecularly characterized at the *Pik* locus, which is located on the distal end of the long arm of chromosome 11 (Chen et al. 2015; Campbell et al. 2004; Ashikawa et al. 2008; Yuan et al. 2011; Zhai et al. 2011; Hua et al. 2012; Ashikawa et al. 2012; Zhai et al. 2014). It is worth noting that many *R*-gene alleles are extremely sequence-related to each other. For example, *Pik-1*, one of two NBS-LRR genes at the *Pik* locus in Kusabue, differs from its allele *Pik1-KA* in Kanto51 by only four nucleotides confined in the region encoding the NBS domain (Zhai et al. 2011; Ashikawa et al. 2012). Other NBS-LRR genes, *Pik-2* and *Pik2-KA*, are even identical to each other (Zhai et al. 2011; Ashikawa et al. 2012). A similar scenario was also observed at the *Pish* locus (Takahashi et al. 2010).

Several approaches were employed for the identification of novel *R* genes or alleles of known *R* loci, such as map-based cloning, allele mining and genome-wide association study (GWAS). Recently, 97 loci associated with rice blast resistance were identified using the GWAS approach (Kang et al. 2015). By combining the RNA interference (RNAi) approach, the candidate gene in LABR_64 corresponding to resistance to all five isolates was validated and confirmed to be an allele of *Pi5*. In addition to the traditional gene-linked markers (Wang et al. 1994; Fjellstrom et al. 2006; Hayashi et al. 2004; Thakur et al. 2014), gene-specific or diagnostic markers were recently reported for the identification of novel blast *R* genes or alleles in diverse germplasm, such as *Pi54* (Ramkumar et al. 2011), *Pikm* (Costanzo and Jia, 2010) and *Pike* (Chen et al. 2015).

The *Pi2/9* locus located on the short arm of chromosome 6 proximal to the centromere was reported to harbor at least eight functional alleles from different donor varieties (Su et al. 2015; Qu et al. 2006; Zhou et al. 2006; Deng et al. 2006; Jeung et al. 2007; Wang et al. 2012; Jiang et al. 2012). Analyses of genetic diversity of the *Pi2/9* homologues in cultivar and wild rice species revealed that the *Pi2/9* homologues were subjected to strong diversifying selection (Zhou et al. 2007; Dai et al. 2010; Liu et al. 2011). Molecular characterization of *Pi2*, *Pi9*, *Piz-t* and *Pi50* revealed that a limited number of sequence variations disproportionately confined within the LRR regions of the encoded *R* proteins mainly determined the distinct recognition specificities of these alleles to different sets of rice blast isolates (Su et al. 2015; Zhou et al. 2006; Qu et al. 2006). Moreover, these alleles are each embedded within a cluster containing multiple sequence-related paralogues at the locus in the respective donor varieties (Su et al. 2015; Zhou et al. 2006; Qu et al. 2006). The feature of the complex organization of highly sequence-related homologues at the *Pi2/9* locus makes it difficult to develop gene-specific molecular markers for the diagnosis and identification of known and novel alleles from diverse germplasm. Intriguingly, contrasting to the absence in susceptible rice varieties, the *Pi2* alleles are exclusively present in the resistant haplotypes (Su et al. 2015; Zhou et al. 2007), prompting us an assumption that the *Pi2* sequence could be targeted for developing markers for allele mining at the *Pi2/9* locus. In this study, we aim to develop a resistant haplotype specific marker at the *Pi2/9* locus and apply it for the identification of novel resistant germplasm containing new *Pi2/9* alleles. The development of introgression lines via marker-assisted backcrossing (MABC) enabling the analysis of resistance spectra of these novel introgression lines against a wide collection of rice blast isolates in the Philippines will be also described.

## Results

### Development and validation of the *Pi2/9* resistant haplotype marker, Pi2/9-RH

**Fig. 1:**
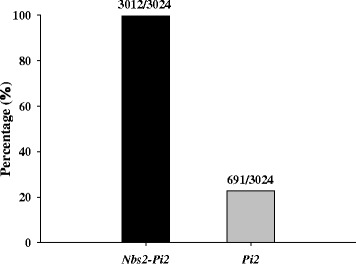
Frequency of *Nbs2-Pi2* and *Pi2* alleles in 3024 rice germplasm accessions. The figure was constructed using the program of Sigmaplot

The availability of medium-depth coverage of genome sequences of 3024 rice accessions allowed us to search the existence of the alleles of *Nbs2-Pi2* and *Nbs4-Pi2* (*Pi2*) in a large rice collection (The 3000 Rice Genomes Project, 2014). It was found that only 12 out of 3024 lines had very limited or even no sequences reads aligning to the promoter of *Nbs2-Pi2* (corresponding to the region of chromosome 6 at the position: 10,380,244–10,381,506 bp in pseudomolecule 1.0 of MSU Rice Genome Annotation Project Release 7-RGAP 7.0, http://rice.plantbiology.msu.edu), suggesting that almost all the sequenced accessions contain the alleles of *Nbs2-Pi2* (Fig. 1). On the contrary, only 691 out of 3024 lines were found to have sequence reads matching the promoter of *Pi2* [corresponding to the region of the *Pi2* locus (Genbank accession no. DQ352453) at the position: 72,301–73,620 bp], indicating that about 23% of the rice accessions contain the alleles of *Pi2* (Fig. 1). The disproportionate distribution pattern of *Nbs2-Pi2* and *Pi2* alleles in 3 K genomes prompted us to further investigate whether the existence of the *Pi2-*unique sequence can infer to the existence of functional *Pi2/9* alleles in the diverse germplasm. A unique sequence fragment corresponding to the promoter and a portion of the first intron of *Pi2* were selected as the region for the development of a resistant haplotype specific marker so called Pi2/9-RH for the PCR screening. Two pairs of primers (Pi2/9-DF1/DR1 and Pi2/9-DF2/DR2) were synthesized and optimized with excellent PCR amplification efficiency (Table 1). Because of the close locations of these two primer pairs, only Pi2/9-DF1/DR1 was used as Pi2/9-RH in this study.

**Table 1:** Primers used in this study

Primer name	Primer sequence (5′ to 3′)	Purpose	Expected Size (bp)
Pi2/9-DF1	CTTGACATCCAAACCGCACC	For the development of the marker Pi2/9-RH	1172
Pi2/9-DR1	TAGGCCTAGCCAATTTTTGCC		
Pi2/9-DF2	CAGACGCTGCCGAAGGCTGC	For the development of the marker Pi2/9-RH	1512
Pi2/9-DR2	CAATAGTTGCTGATTCCTGAGC		
5UTR-F	CTTGAAGGGAGAGTCGAACG	For the cloning of full-length cDNA of *Pi2/9* homologues	3235
3UTR-R	GCCTCATTGATCATCATGCC		

We firstly used LTH-derived International Rice Research Institute (IRRI) bred blast resistant lines (IRBLs) containing different *R* genes for the analysis. Four IRBLs containing *Pi2*, *Pi9*, *Piz-t* and *Piz* were each resolved with a PCR amplicon at the expected size using Pi2/9-RH. On the contrary, no PCR amplification was resolved in other IRBLs containing non-*Pi2/9* genes and five susceptible rice varieties (Additional file 1: Figure S1). We further tested the existence of Pi2/9-RH in five rice panels. In the panel of 50 accessions showing susceptibility to at least one of two isolates (Panel IV) from the rice 2 K panel (http://ricephenonetwork.irri.org/diversity-panels), only 6% of 50 rice lines showed positive PCR amplification by Pi2/9-RH (Table 2). It was also found that only 8% of 50 IRRI released varieties and advanced breeding lines (Panel V) showed positive PCR amplification. On the contrary, the resistant rice lines obtained by screening against different number of rice blast isolates showed 24.4% and 45.5% frequency of positive PCR amplification, respectively, in Panel III and I (Table 2). Moreover, the rice lines resistant to more isolates displayed a higher frequency than the ones resistant to fewer isolates (45.5% in Panel I versus 24.4% in Panel III) (Table 2 and Additional file 2: Table S1). These data suggested a positive correlation between the frequency of the existence of Pi2/9-RH and resistance to rice blast in the rice panels.

**Table 2:** Frequency of *Pi2* orthologues in different rice panels diagnosed by using the primer pair Pi2/9-RH

Rice panel^a^	Number of accessions assessed	Number of positive accessions	Frequency (%)
I^b^	55	25	45.5
III^c^	156	38	24.4
IV^d^	50	4	8.0
V^e^	50	3	6.0

^a^The information of rice accessions and reactions to different rice blast isolates is listed in Table S1. ^b^Panel I consisted of 55 lines showing resistance against 4 isolates. A16 was not counted in the frequency calculation due to the amplicon with an unexpected size. ^c^Panel III consisted of 156 lines showing resistance against two rice blast isolates. ^d^Panel IV consisted of 50 randomly selected lines from the IRRI rice 2 K diversity panel. ^e^Panel V consisted of 50 IRRI-released varieties and advanced lines

### Identification of five *Pi2/9* haplotype groups each containing different *Pi2/9* homologues in resistant rice germplasm

The identification of 26 Pi2/9-RH positive lines in Panel I (Additional file 2: Table S1) promoted us to investigate whether they contain known or novel *Pi2/9* homologues except A16 which was resolved with a PCR amplicon of a larger size (Additional file 1: Figure S2). We attempted the cloning of full-length coding sequences (CDSs) using conserved primers 5UTR-F/3UTR-R (Table 1) and proceeded for sequencing. Cluster analysis based on the partial sequence of the CDSs resulted in five unique haplotypes each containing different *Pi2/9* homologues which were named *Pi2/9-A5*, *−A15*, *−A42*, *−A53*, and *-A54* (Table 3). Interestingly, *Pi2/9-A5* and -*A54* were identical in sequence to *Piz-t* and *Pi2*, respectively, which was further confirmed by sequencing the entire CDS (Table 3). On the contrary, *Pi2/9-A15*, −*A42* and -*A53* each contained sequence differences from any of the known *Pi2/9* alleles, suggesting these haplotypes contained novel *Pi2/9* homologues.

**Table 3:** Clustering of 25 Pi2/9-RH positive rice lines by sequencing the *Pi2/9* homologues

*Pi2/9* homologues	Number of accessions carrying the same homologues	IRGC accession number	Varietal group	Country of origin	Remark
*Pi2/9-A5*	5	121299	*Indica*	Thailand	Identical to *Piz-t* in nucleotide sequence
		121383	*Indica*	Lao PDR	
		121392	*Indica*	Bangladesh	
		121753	*Indica*	Philippines	
		121898	*Aus/boro*	India	
*Pi2/9-A15*	4	121434	*Indica*	China	
		121607	*Indica*	India	
		121611	*Indica*	Chinese Taiwan	
		121660	*Indica*	Philippines	
*Pi2/9-A42*	14	121315	*TropJap*	Colombia	
		121490	*TropJap*	Bolivia	
		121536	*TropJap*	Côte d’Ivoire	
		121632	*Indica*	Colombia	
		121698	*TropJap*	Philippines	
		121699	*TropJap*	Madagascar	
		121730	*TropJap*	Madagascar	
		121744	*TropJap*	France	
		121749	*TropJap*	Philippines	
		121755	*TropJap*	Philippines	
		121762	*TropJap*	Ghana	
		121764	*TropJap*	Côte d’Ivoire	
		121804	*Indica*	Colombia	
		121805	*TropJap*	Colombia	
*Pi2/9-A53*	1	121884	*Indica*	Vietnam	
*Pi2/9-A54*	1	121888	*Indica*	Panama	Identical to *Pi2* in nucleotide sequence

*IRGC* International Rice Genebank Collection, *TropJap* tropical japonica

It was found that these 25 rice lines originated from different countries in Asia, Africa, Europe and South America, representing a wide geographic distribution in the world (Table 3). Some *Pi2/9* alleles were identified in multiple rice lines from different regions, for example, *Pi2/9-A42* was present in 14 rice lines. Moreover, 12 out of 14 rice lines belonged to the subgroup of tropical japonica, representing a disproportional distribution of *Pi2/9-A42* in different rice subgroups (Table 3).

### Introgression lines containing 3 novel *Pi2/9* haplotypes showed broad-spectrum resistance against rice blast isolates in the Philippines

To validate the association between the *Pi2/9* haplotypes and resistance against rice blast, we generated BC_3_F_3_ introgression lines of *Pi2/9-A15*, *Pi2/9-A42* and *Pi2/9-A53* in the background of the susceptible variety CO39 via MABC using the marker of Pi2/9-RH and named the derived monogenic lines as IR126181, IR126183 and IR126184, respectively. These 3 introgression lines together with IRBLs were assessed to a set of 34 CO39-virulent isolates from a diverse collection in the Philippines for the resistance spectrum analysis. As Table 4 indicated, both IR126181 (*Pi2/9-A15*) and IR126184 (*Pi2/9-A53*) were resistant to all isolates as observed in IRBL9-W (*Pi9*). IR126183 (*Pi2/9-A42*) was resistant to 24 isolates and susceptible to 4 isolates. Intriguingly, IR126183 showed partial resistance to four isolates that developed typical type-3 lesions on the leaves (Table 4 and Additional file 1: Figure S3). Out of these 34 isolates, 2 and 23 were virulent to IRBL-z5[CO] (*Pi2*) and IRBL-zt[CO] (*Piz-t*), respectively (Table 4).

**Table 4:** Disease reaction patterns of the introgression lines IR126181 (*Pi2/9-A15*), IR126183 (*Pi2/9-A42*), IR126184 (*Pi2/9-A53*), IRBL-z5[CO] (*Pi2*), IRBL-zt[CO] (*Piz-t*) and IRBL-9W (*Pi9*) and two susceptible lines, CO39 and Lijiangxintuanheigu (LTH), against 34 *M. oryzae* isolates^a^

Isolate	CO39	New introgression lines^b^			*Pi2/9* known allele introgression lines^c^			LTH
		IR126181 (*Pi2/9-A15*)	IR126183 (*Pi2/9-A42*)	IR126184 (*Pi2/9-A53*)	IRBL-z5[CO] (*Pi2*)	IRBL-zt[CO] (*Piz-t*)	IRBL-9 W (*Pi9*)	
BN111	S	R	R	R	R	S	R	S
BN209	S	R	NA	R	S	S	R	S
CA41	S	R	R	R	R	R	R	S
CA89	S	R	PR	R	R	S	R	S
IK81–25	S	R	R	R	R	S	R	S
JMB8401	S	R	R	R	R	S	R	S
JMB840610	S	R	S	R	R	R	R	S
M36–1–3-10-1	S	R	R	R	R	R	R	S
M39–1–3-8-1	S	R	R	R	R	R	R	S
M64–1–3-9-1	S	R	S	R	R	R	R	S
PO6–6	S	R	R	R	R	S	R	S
V86010	S	R	S	R	R	R	R	S
5008–3	S	R	R	R	R	S	R	S
5092–3	S	R	R	R	R	S	R	S
5167–1	S	R	NA	R	S	R	R	S
6161–1	S	R	R	R	R	S	R	S
9126–1	S	R	R	R	R	S	R	S
9244–3	S	R	R	R	R	S	R	S
9406–3	S	R	R	R	R	S	R	S
9475–1-3	S	R	R	R	R	S	R	S
9482–1-3	S	R	S	R	R	R	R	S
9497–3	S	R	PR	R	R	S	R	S
IBN008	S	R	R	R	R	S	R	S
IBN028	S	R	R	R	R	S	R	S
MO15–1	S	R	R	R	R	S	R	S
MO15–6	S	R	PR	R	R	S	R	S
MO15–19	S	R	R	R	R	R	R	S
MO15–21	S	R	R	R	R	S	R	S
MO15–24	S	R	R	R	R	R	R	S
MO15–27	S	R	PR	R	R	S	R	S
MO15–110	S	R	R	R	R	S	R	S
MO15–125	S	R	R	R	R	R	R	S
MO15–226	S	R	R	R	R	S	R	S
MO15–244	S	R	R	R	R	S	R	S

^a^R indicates resistance, PR indicates partial resistance, S indicates susceptibility and NA indicates not available. Resistance evaluations are based on the 0–5 scale of the Standard Evaluation System. ^b^The three new introgression lines are all in the CO39 genetic background. ^c^IRBL-z5[CO] and IRBL-zt[CO] are introgression lines of *Pi2* and *Piz-t* in the genetic background of CO39; IRBL9-W is the introgression line of the *Pi9* gene in the genetic background of LTH

**Fig. 2:**
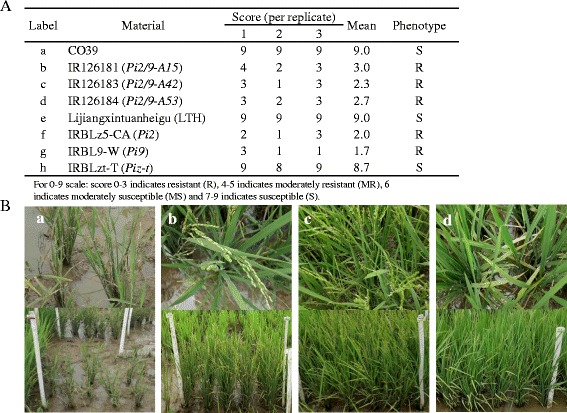
Field assessment of the introgression lines IR126181 (*Pi2/9-A15*), IR126183 (*Pi2/9-A42*), IR126184 (*Pi2/9-A53*), IRBLz5-CA (*Pi2*), IRBL9-W (*Pi9*) and IRBLzt-T (*Piz-t*) together with the susceptible control CO39 and LTH. **a** Scoring of the introgression lines in the field with three replicates for each line. **b** Photograph of the disease reaction of introgression lines of *b* IR126181, *c* IR126183, *d* IR126184 and *a* susceptible control CO39

To evaluate the resistance of these three introgression lines in the field, we tested them with different IRBLs in a field hot spot in Bohol, the Philippines in 2016. They all showed strong resistance whereas LTH and CO39 showed high susceptibility to blast in the field (Fig. 2). Similar to these 3 introgression lines, IRBLz5-CA (*Pi2*) and IRBL9-W showed strong resistance. On the contrary, IRBLzt-T (*Piz-t*) was susceptible. Taken together, we postulated that these 3 introgression lines IR126181, IR126183 and IR126184 controlled broad-spectrum resistance against rice blast isolates in the Philippines (Fig. 2).

### The resistance of introgression lines was associated with the *Pi2/9* haplotypes

To validate whether the resistance of the introgression lines was associated with the *Pi2/9* haplotypes, each BC_3_F_2_ population used for the advancement of BC_3_F_3_ introgression lines was proceeded with genetic analysis. As Table 5 listed, 550 out of 720 progenies of the BC_3_F_2_ population of *Pi2/9-A15* were resistant whereas 170 progenies were susceptible to the isolate MO15–21, displaying an expected 3:1 ratio of resistance versus susceptibility. The same ratio was also observed for resistant versus susceptible progenies of the BC_3_F_2_ population of *Pi2/9-A53* against the isolate 5167–1. These data indicated that the resistance of *Pi2/9-A15* and *Pi2/9-A53* introgression lines was each controlled by a single genetic locus. For the case of *Pi2/9-A42*, an expected 15:1 ratio of resistance versus susceptibility was observed in the 463 BC_3_F_2_ progenies to the isolate MO15–21, indicating that the resistance was controlled by two individual genetic loci. To further validate the linkage of resistance to the *Pi2/9* locus, all the susceptible plants for each BC_3_F_2_ population underwent genotyping with Pi2/9-RH. None of the susceptible progenies produced the positive PCR amplicon (Table 5), indicating that the resistance could be tightly linked with the marker of Pi2/9-RH. We thus speculated that the *Pi2/9* locus or tightly linked one was likely responsible for the resistance observed in the introgression lines of *Pi2-A15*, *Pi2-A42* and *Pi2-A53*.

**Table 5:** Linkage analysis between blast resistance and the *Pi2/9* haplotypes in introgression lines

Isolates	BC_3_F_2_ population	Number of progenies			Chi-square test			Number of susceptible progenies using Pi2/9-RH	
		R	S	Total	Expected ratio (R:S)	χ^2^	P	Positive	Negative
MO15–21	CO39/A15	550	170	720	3:1	0.741	0.389	0	170
MO15–21	CO39/A42	433	30	463	15:1	0.042	0.838	0	30
5167–1	CO39/A53	571	195	766	3:1	0.085	0.770	0	195

*R* resistance, *S* susceptibility

## Discussion

### An integrated approach combines germplasm screening by resistant haplotype specific marker, sequencing of full-length cDNA and development of monogenic lines for efficient identification of resistant germplasm containing putatively functional *Pi2/9* alleles

Exploration of novel blast *R* genes or alleles is a continuous effort to ensure the selection of the most effective ones for breeding resistant rice varieties. In addition to the traditional map-based cloning strategy, several approaches were successfully developed for the fast characterization of *R* genes, such as GWAS (Kang et al. 2015), MutMap-Gap (Takagi et al. 2013) and *R*-gene analog (RGA)-based linkage analysis coupled with mutant characterization (Okuyama et al. 2011). Thanks to the advances in sequencing technology and increasing genome information, allele mining provides a powerful and economical approach for the identification of novel alleles (Bhullar et al. 2010). For example, nine new alleles of *Pi54* containing sequence differences from known ones were identified in 885 Indian rice genotypes via allele mining (Vasudevan et al. 2015). Functional validation of alleles containing genetic variations followed by identification is another key step in allele mining, which is usually carried out by gene complementation tests, silencing or knockout approaches. For example, the function of *Pid3-A4*, a novel allele of *Pid3* identified in the common wild rice A4 by referring to the sequence of *Pid3* in cultivated rice, was validated by generation and resistance assessment of *Pid3-A4* transgenic plants against rice blast isolates (Lv et al. 2013). However, the fact that rice blast *R* genes are often organized in complexes and are highly similar in sequence from one another hinders the efficient identification and functional validation of candidate genes in the process of allele mining. In this regard, development of diagnostic markers becomes a prerequisite for efficient screening of known alleles of *R* genes in diverse germplasm. The *Pi2/9* locus is one of the complex loci which were extensively investigated for the identification of different alleles in diverse germplasm by using various gene-linked or gene-specific markers including simple sequence repeat (SSR), insertion/deletion (InDel), and cleavage amplified polymorphisms (CAPS) markers (Jiang et al. 2015; Liu et al. 2002; Hayashi et al. 2006; Zhu et al. 2012; Tian et al. 2016). These markers were used either for the identification of particular alleles in contained populations or for the diagnosis of known alleles in different germplasm. For the latter case, however, it is really necessary to validate the correlation between the existence of know alleles and the polymorphic pattern of markers, particularly for gene linked SSR markers, due to the extreme sequence similarity among different functional and nonfunctional alleles at the *Pi2/9* locus (Su et al. 2015; Zhou et al. 2007). Moreover, those markers have limited application in the identification of novel alleles at the *Pi2/9* locus. It is indeed that, similarly to the *Pi2/9* gene family, most rice blast *R* genes are classified into Type-II *R* gene family whose members differ by a limited number of point mutations (Luo et al. 2012). Thus, it is valuable to develop an approach to quickly identify germplasm containing novel *Pi2/9* alleles before the extensive molecular characterization. In this study, we developed an integrated approach for the efficient identification of germplasm showing broad-spectrum resistance against blast resistance which was likely conferred by novel *Pi2/9* alleles or their tightly linked genetic loci. First, the resistant haplotype specific marker Pi2/9-RH was developed and used for the identification of germplasm containing putative *Pi2/9* alleles for further functional characterization. Different from other allele-specific markers for known alleles, Pi2/9-RH can also be applied for the identification of novel alleles. Then, sequences of *Pi2/9* homologues in Pi2/9-RH positive lines helped the clustering of identical haplotypes to avoid redundant functional characterization. Last, the generation of monogenic rice lines each containing individual novel *Pi2/9* haplotypes allowed the functional validation and spectrum analysis, demonstrating an alternative approach for the functional characterization of the *Pi2/9* locus containing resistant alleles. Moreover, the derived new monogenic lines can be freely distributed for field tests in different countries, providing valuable materials for resistance spectrum/frequency analysis of novel *Pi2/9* alleles against rice blast under different environments. Taken together, the integrated approach presented herein provides an efficient scheme for identifying novel *Pi2/9* alleles, which could also be applied to allele mining at other *R*-gene loci.

### The *Pi2/9* locus harbors promising alleles conferring broad-spectrum resistance against rice blast

It has been documented that the resistance spectra of different *R* genes are mainly determined by the frequency of cognate *Avr* genes in the rice blast pathogen population. Therefore, the mechanisms underlying the recognition of their cognate *Avr* genes determine the evolution of resistance spectra controlled by different *R*-gene alleles at the same locus. In general, two main scenarios were demonstrated in the rice and rice blast phytopathosystem with respect to *R/Avr* recognition. The first scenario captures a step-wise arms race between different *R*-gene alleles and different *Avr*-gene haplotypes. For example, the *Pik* locus harbors multiple *Pik* alleles activating resistance to rice blast by recognizing different *AvrPik* haplotypes containing only one to three amino acid changes from one another (Yoshida et al. 2009; Kanzaki et al. 2012; Wu et al. 2014). In this scenario, new *R*-gene alleles containing sequence differences from known ones are not assumed to necessarily alter the resistance spectrum, thus limiting the value for identifying novel *R*-gene alleles. On the contrary, the *Pi2/9* locus consists of multiple alleles mediating resistance against rice blast by recognizing sequence-unrelated *Avr* genes, illustrating a contrasting scenario in the rice and rice blast phytopathosystem. For example, *Piz-t* and *Pi9* recognize sequence-unrelated *AvrPiz-t* and *AvrPi9*, respectively, although *Piz-t* and *Pi9* are highly sequence-related to each other (Zhou et al. 2006, Qu et al. 2006; Wu et al. 2015; Li et al. 2009). This suggests more chances to identify novel alleles conferring distinct resistance spectra at *Pi2/9* loci. Indeed, another two alleles (*Pi2* and *Pi50*) were found to each control resistance against diverse sets of isolates via recognition of different *Avr* genes other than *AvrPi9* and *AvrPiz-t* (Su et al. 2015). More significantly, *Pi2/9* alleles were reported to confer broad-spectrum resistance against rice blast in different rice-growing areas worldwide (Liu et al. 2002; Zhu et al. 2012). A recent study on large-scale germplasm screening for broad-spectrum resistance sources revealed that half of the 289 broad-spectrum blast-resistant genotypes harbored the *Pi2* locus validated by an STS marker (Vasudevan et al. 2014). In this study, we identified 3 haplotypes containing 3 novel *Pi2/9* homologues. The derived monogenic lines each controlled broad-spectrum resistance against diverse isolates. These data suggests that the *Pi2/9* locus is likely responsible for the resistance against rice blast, which was also supported by the genetic analysis. However, the possibility that other *R* gene loci tightly linked to the *Pi2/9* locus conferred the resistance observed in these monogenic lines could not be excluded due to relatively low resolution of genetic mapping. Fine mapping or gene complementation tests of the candidate genes should be able to further clarify the function of novel *Pi2/9* alleles in these 3 haplotypes.

It is worth noting that blast *R* genes/alleles genetically identified from different donors in different geographic regions could be identical in sequence. For example, *Pi25* from Guimei 2 is identical to *Pid3* from Digu in protein sequence (Chen et al. 2011; Shang et al. 2009). It was also found that some *R* genes/alleles from different donors differing by limited sequence variations conferred the same resistance spectrum by recognizing identical *Avr* genes. In this regard, these different *R* genes/alleles can be considered identical based on their resistance spectrum. For example, *Pik1-KA* from Kanto51 differs from *Pik-1* from Kusabue by only four nucleotide sequences and *Pik2-KA* and *Pik-2* are identical to each other. Both *Pik1-KA* and *Pik* were characterized to control identical resistance spectra by recognizing the same sets of *AvrPik* haplotypes (Zhai et al. 2011; Ashikawa et al. 2012). In this study, we found that *Pi2/9-A42*, *Pi2/9-A15* and *Piz-t* were, respectively, identified in 14, 4 and 5 rice lines from different countries, demonstrating a wide distribution of the same *R* genes/alleles in diverse germplasm. It is thus reasonable to assume that the *R* genes/alleles in the same locus identified in different landraces described by different research programs could be identical in sequence or functionality, which raises a concern regarding a systemic nomenclature of rice blast *R* genes before molecular characterization.

## Conclusion

We identified three resistance germplasm containing novel *R* genes at or tightly linked to the *Pi2/9* locus which conferring broad-spectrum resistance against rice blast. The marker Pi2/9-RH which developed from the conserved 5′ portion of the *Pi2* sequence could be widely used as a diagnostic marker for the quick identification of resistance donors containing functional *Pi2/9* alleles or unknown linked *R* genes. The development of three new introgression lines containing the *Pi2/9* introgression segment may play an important role in disease assessment and rice blast resistance breeding.

## Methods

### Plant materials and *M. oryzae* isolates

The rice 2 K panel consisting of 1400 rice accessions was obtained from the International Rice Genebank Collection (IRGC) of IRRI, Philippines. Fifty IRRI varieties, 30 IRBLs in the genetic background of Lijiangxintuanheigu (LTH), 2 IRBLs [IRBLz5-CA (CO) and IRBLzt-IR56 (CO)] in the genetic background of CO39, and other rice varieties including CO39, 9311, LTH, Taipei309 and Nipponbare used in this study were maintained at IRRI (Additional file 2: Table S1 and Table S2). Thirty-six *M. oryzae* isolates collected from four provinces (Laguna, Camarines Sur, Batangas and Bohol) of the Philippines in different years used in this study were maintained in the rice blast isolate collection at IRRI (Additional file 2: Table S3).

### Disease evaluation in greenhouse and field

For greenhouse inoculation, 14-day-old rice seedlings (3–4 leaves) were sprayed with spore suspension (1 × 10^5^ spores/mL) of individual rice blast isolates. The lesion types on the leaves were scored 7 days post-inoculation using the 0–5 standard scale (Campbell et al. 2004). In this study, plants having lesion scores of 0, 1 and 2 were considered as resistant (R), of 3 were considered as partial resistance (PR), and of scores of 4 and 5 were considered as susceptible (S). For the field evaluation, different rice lines in a 60 cm × 30 cm plot were grown in a randomized complete block design with three replications at the hotspot experimental site in Ubay, Bohol, the Philippines. Disease evaluation was carried out at 40 days after transplanting when the blast disease reached to the peak. Disease severity was scored by following the 0–9 standard scale developed by IRRI (IRRI 2002).

### Screening for resistant rice germplasm accessions

Three rounds of screening were employed to identify resistant rice germplasm in the 2 K panel. First, two *M. oryzae* isolates (CA89 and M64–1–3-9-1) were used to inoculate all the 1400 rice accessions in greenhouse, which led to the identification of 356 resistant accessions (Additional file 2: Table S1). Out of the 356 resistant accessions, 200 were randomly selected for the second round of screening with another two isolates (JMB8401 and M101–1–2-9-1) whereas another 156 lines were used for determining the frequency of *Pi2* orthologues (Additional file 2: Table S1). Fifty-five out of 200 lines were found either resistant or partially resistant to all 4 isolates (Additional file 2: Table S1). These 55 resistant lines were further inoculated with 5 more isolates (9239–4, CA41, IK81–25, M36–1–3-10-1 and M39–1–3-8-1) for the analysis of resistance spectrum (Additional file 2: Table S1).

### DNA extraction and PCR amplification

Genomic DNA of all the rice accessions was extracted from the leaf samples by using the CTAB DNA extraction method. PCR amplification using the prime pairs for Pi2/9-RH (Table 1) was carried out using the following profile: initial DNA denaturation at 95 °C for 5 min; followed by 35 cycles of denaturation at 98 °C for 10 s, annealing at 58 °C for 30 s and extension at 72 °C for 80s; and final extension at 72 °C for 5 min.

### RT-PCR amplification and DNA sequencing

Total RNA was isolated from the leaf tissue by using TRIZOL Reagent (Life Technologies) according to the manufacturer’s instructions. In brief, 100 mg of leave was ground using a mortar and pestle with liquid nitrogen, and the powder was suspended in 1 ml of Trizol. Following by 10 min of incubation, 0.2 mL of chloroform was added, and samples were mixed manually for 20 s and then incubated for 4 min. After centrifugation (rcf 11,000 g) for 15 min at 4 °C, the aqueous layer was retrieved and mixed with 0.25 mL of 3 M sodium acetate, pH 5.2, and 0.25 mL of isopropanol. A pellet was obtained by centrifugation (rcf 11,000 g) and then washed twice with 75% ethanol. After treatment by DNaseI (DNA-free TM Kit, Ambion), purified RNA was proceed with reverse transcription using the Super Script III First-strand kit (Invitrogen) to obtain first-strand cDNA. RT-PCR was further preceded by using the primer pair 5UTR-F/3UTR-R for the amplification of full-length cDNA of *Pi2/9* homologues (Table 1). The 3′ portions of the *Pi2/9* homologues containing the sequence variations from one another were sequenced at MACROGENE Company (Korea).

### Bioinformatics analysis of *Pi2* and *Nbs2-Pi2* orthologue in 3 K genomes

The promoter sequences of *Pi2* and *Nbs2-Pi2* were aligned to the reference genome sequence (Nipponbare RGAP 7.0, http://rice.plantbiology.msu.edu) to check whether these sequences were in the reference genome or not (Additional file 1: Figure S4). After confirming that the promoter sequences of *Pi2* and *Nbs-Pi2* were not in the reference genome, we aligned all sequencing reads from the 3 K panel to the reference genome to collect the unmapped sequences. The unmapped reads were used in identifying the varieties that contain the *Pi2* and *Nbs2-Pi2* promoter sequences. The unmapped reads of the 3 K panel were aligned separately from the *Pi2* and *Nbs2-Pi2* promoter sequences as the reference genome using the BWA-PICARD-SAMTOOLS pipeline (Additional file 1: Figure S4). This pipeline was used to generate the alignment file (bam), and then a custom perl script was used to detect the presence of the *Pi2* and *Nbs2-Pi2* promoter sequences in each variety of the 3 K panel (Additional file 1: Figure S4).

### Computational analysis

Chi square test was conducted for the segregation of resistant and susceptible plants. The DNA sequences were edited with Sequencher (http://www.genecodes.com) and the edited sequences were aligned with Clustal Omega (http://www.ebi.ac.uk/Tools/msa/clustalo/).

## Additional files

Additional file 1: Figure S1. PCR amplification results of the 30 IRBLs and five rice cultivars using primer pair Pi2/9-DF1/DR1 (Pi2/9-RH). LTH, Lijiangxintuanheigu. IRBL, IRRI-bred blast-resistant lines. **Figure S2.** PCR amplification results of the 26 candidate resistant accessions using primer pair Pi2/9-DF1/DR1 (Pi2/9-RH). CO39 was used as a negative control while the *Pi2* introgression line (IRBLz5-CA) was used as a positive control. **Figure S3.** Disease reaction of introgression line IR126183 (*Pi2/9-A42*) inoculated with the isolates 9244–3, M015–6 and 9482–1-3. R, resistance; PR, partial resistance; S, susceptibility; “+” indicates the PCR result is positive by Pi2/9-DF1/R1. **Figure S4.** Methods used for bioinformatics analysis of Pi2 and Nbs2-Pi2 alleles in 3K genomes. (PPTX 595 kb)

Additional file 2: Table S1. Selected rice panels and reaction of rice germplasm accessions against different isolate. **Table S2.** IRRI-bred blast-resistance lines (IRBLs) in the genetic background of Lijiangxintuanheigu (LTH). **Table S3.** Rice blast (*M. oryzae*) isolates used in this study and their collected place and year. (DOCX 83 kb)
