# Supplementary material for: Identification of resistant germplasm containing novel resistance genes at or tightly linked to the Pi2/9 locus conferring broad-spectrum resistance against rice blast
Source: Rice (N Y). 2017 Aug 4;10:37. doi: 10.1186/s12284-017-0176-z (PMC5544663; doi:10.1186/s12284-017-0176-z)
Supplement: Supplementary file 1 — PCR amplification results of the 30 IRBLs and five rice cultivars using primer pair Pi2/9-DF1/DR1 (Pi2/9-RH). LTH, Lijiangxintuanheigu. IRBL, IRRI-bred blast-resistant lines. Figure S2. PCR amplification results of the 26 candidate resistant accessions using primer pair Pi2/9-DF1/DR1 (Pi2/9-RH). CO39 was used as a negative control while the Pi2 introgression line (IRBLz5-CA) was used as a positive control. Figure S3. Disease reaction of introgression line IR126183 (Pi2/9-A42) inoculated with the isolates 9244–3, M015–6 and 9482–1-3. R, resistance; PR, partial resistance; S, susceptibility; “+” indicates the PCR result is positive by Pi2/9-DF1/R1. Figure S4. Methods used for bioinformatics analysis of Pi2 and Nbs2-Pi2 alleles in 3K genomes. (PPTX 595 kb) [file 12284_2017_176_MOESM1_ESM.pptx]

## Slide 1
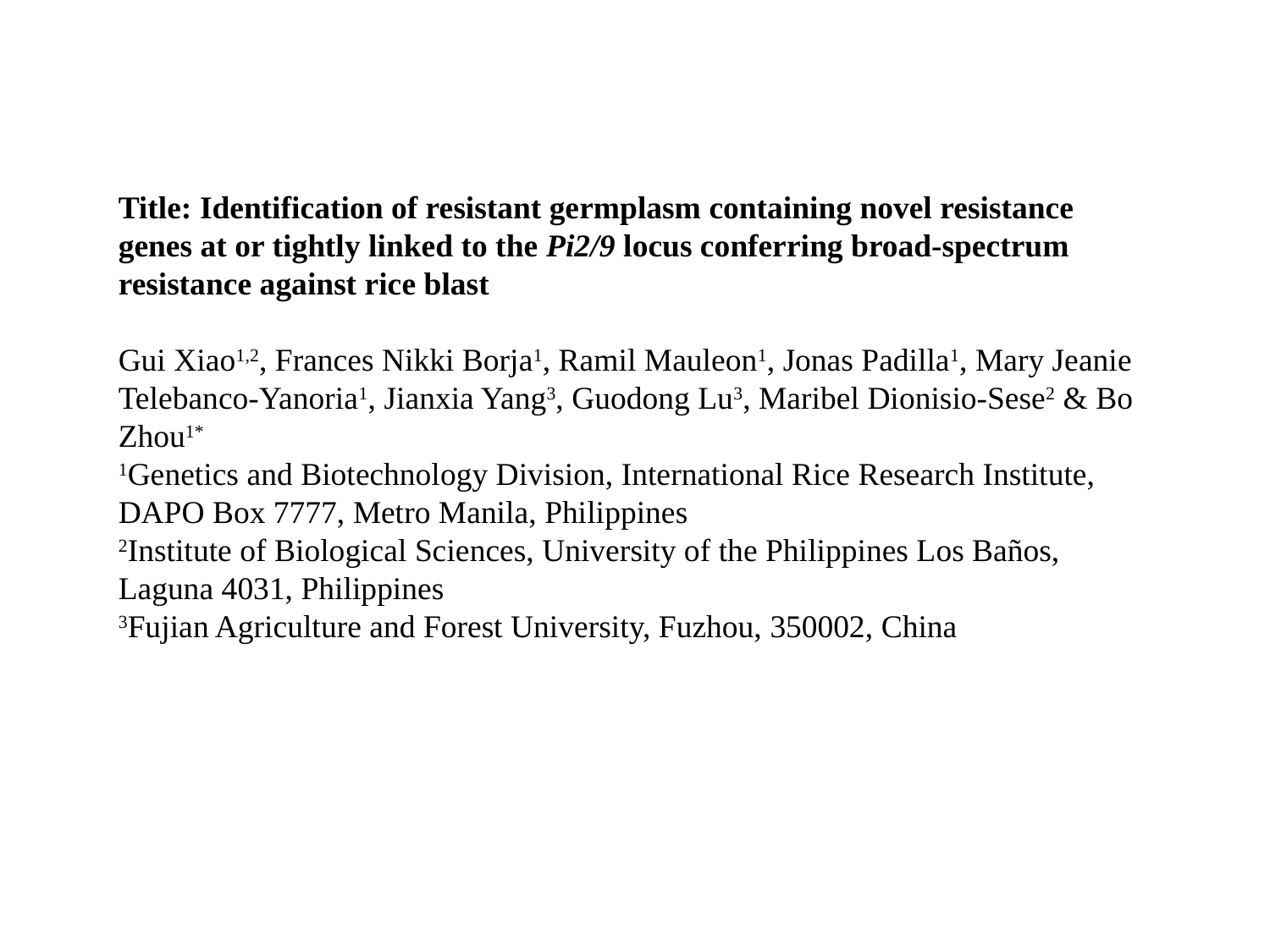

Title: Identification of resistant germplasm containing novel resistance genes at or tightly linked to the Pi2/9 locus conferring broad-spectrum resistance against rice blast
Gui Xiao1,2, Frances Nikki Borja1, Ramil Mauleon1, Jonas Padilla1, Mary Jeanie Telebanco-Yanoria1, Jianxia Yang3, Guodong Lu3, Maribel Dionisio-Sese2 & Bo Zhou1*
1Genetics and Biotechnology Division, International Rice Research Institute, DAPO Box 7777, Metro Manila, Philippines
2Institute of Biological Sciences, University of the Philippines Los Baños, Laguna 4031, Philippines
3Fujian Agriculture and Forest University, Fuzhou, 350002, China

## Slide 2
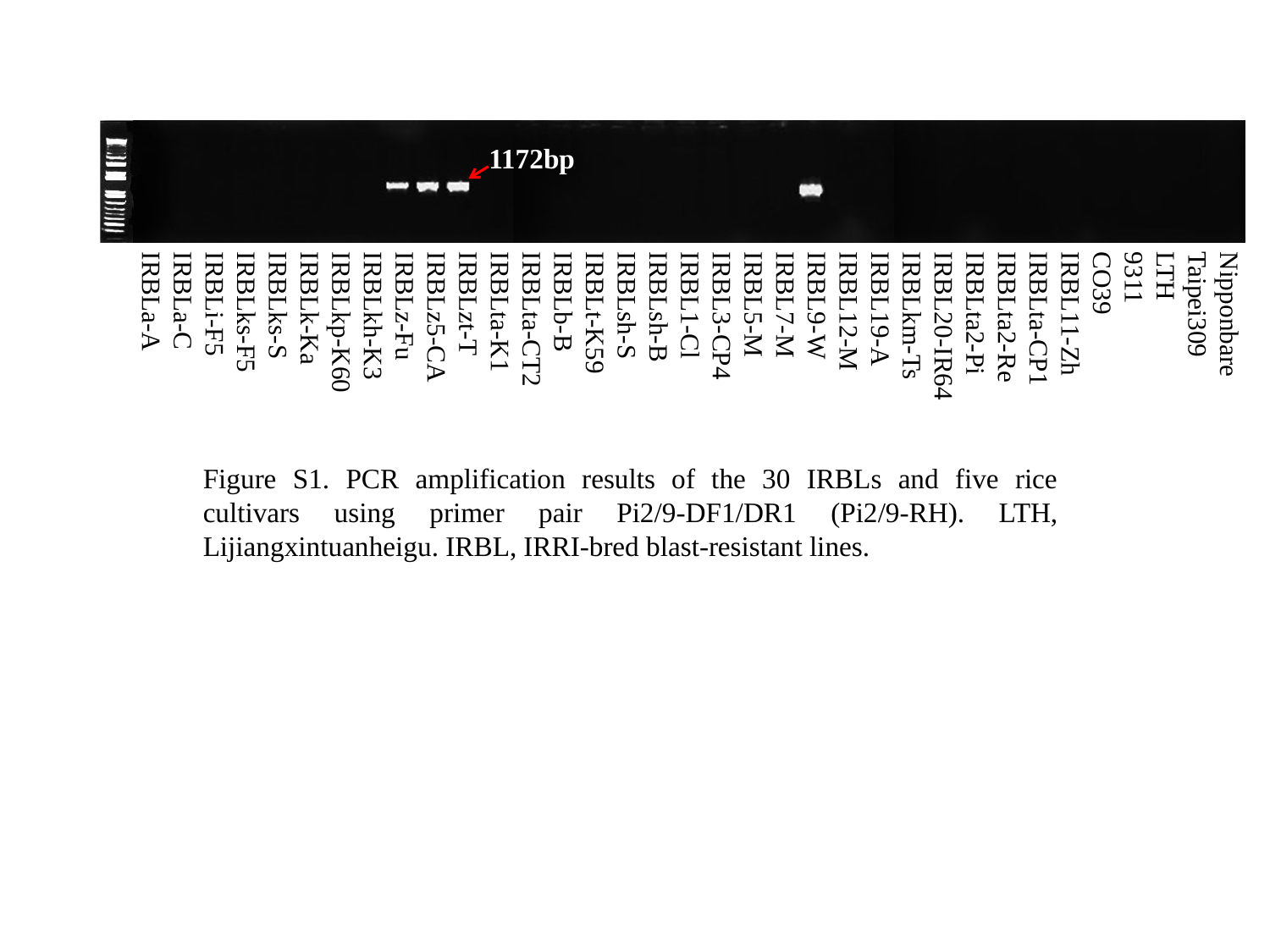

Nipponbare
Taipei309
LTH
9311
CO39
IRBL11-Zh
IRBLta-CP1
IRBLta2-Re
IRBLta2-Pi
IRBL20-IR64
IRBLkm-Ts
IRBL19-A
IRBL12-M
IRBL9-W
IRBL7-M
IRBL5-M
IRBL3-CP4
IRBL1-Cl
IRBLsh-B
IRBLsh-S
IRBLt-K59
IRBLb-B
IRBLta-CT2
IRBLta-K1
IRBLzt-T
IRBLz5-CA
IRBLz-Fu
IRBLkh-K3
IRBLkp-K60
IRBLk-Ka
IRBLks-S
IRBLks-F5
IRBLi-F5
IRBLa-C
IRBLa-A
1172bp
Figure S1. PCR amplification results of the 30 IRBLs and five rice cultivars using primer pair Pi2/9-DF1/DR1 (Pi2/9-RH). LTH, Lijiangxintuanheigu. IRBL, IRRI-bred blast-resistant lines.

## Slide 3
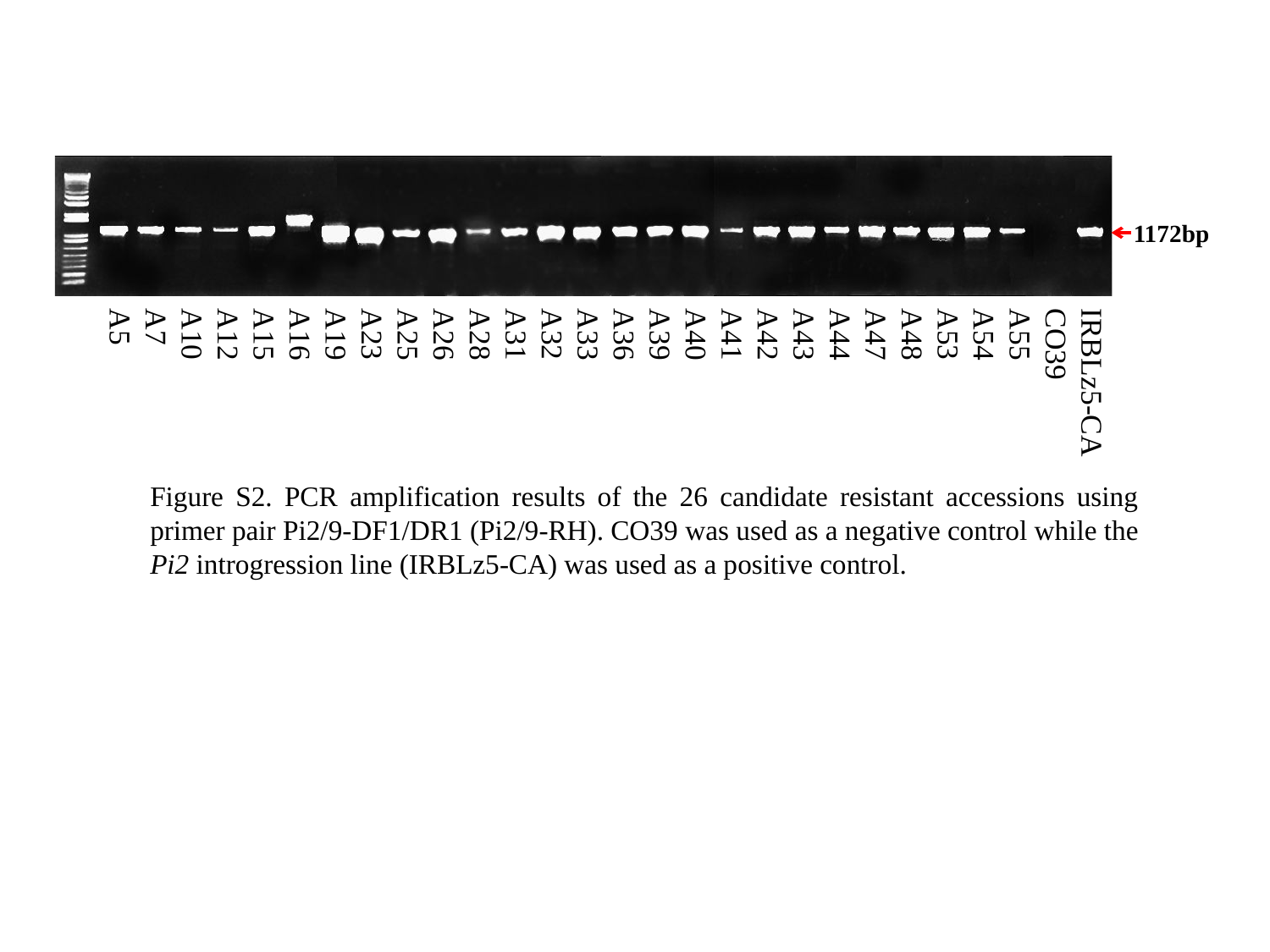

1172bp
IRBLz5-CA
CO39
A55
A54
A53
A48
A47
A44
A43
A42
A41
A40
A39
A36
A33
A32
A31
A28
A26
A25
A23
A19
A16
A15
A12
A10
A7
A5
Figure S2. PCR amplification results of the 26 candidate resistant accessions using primer pair Pi2/9-DF1/DR1 (Pi2/9-RH). CO39 was used as a negative control while the Pi2 introgression line (IRBLz5-CA) was used as a positive control.

## Slide 4
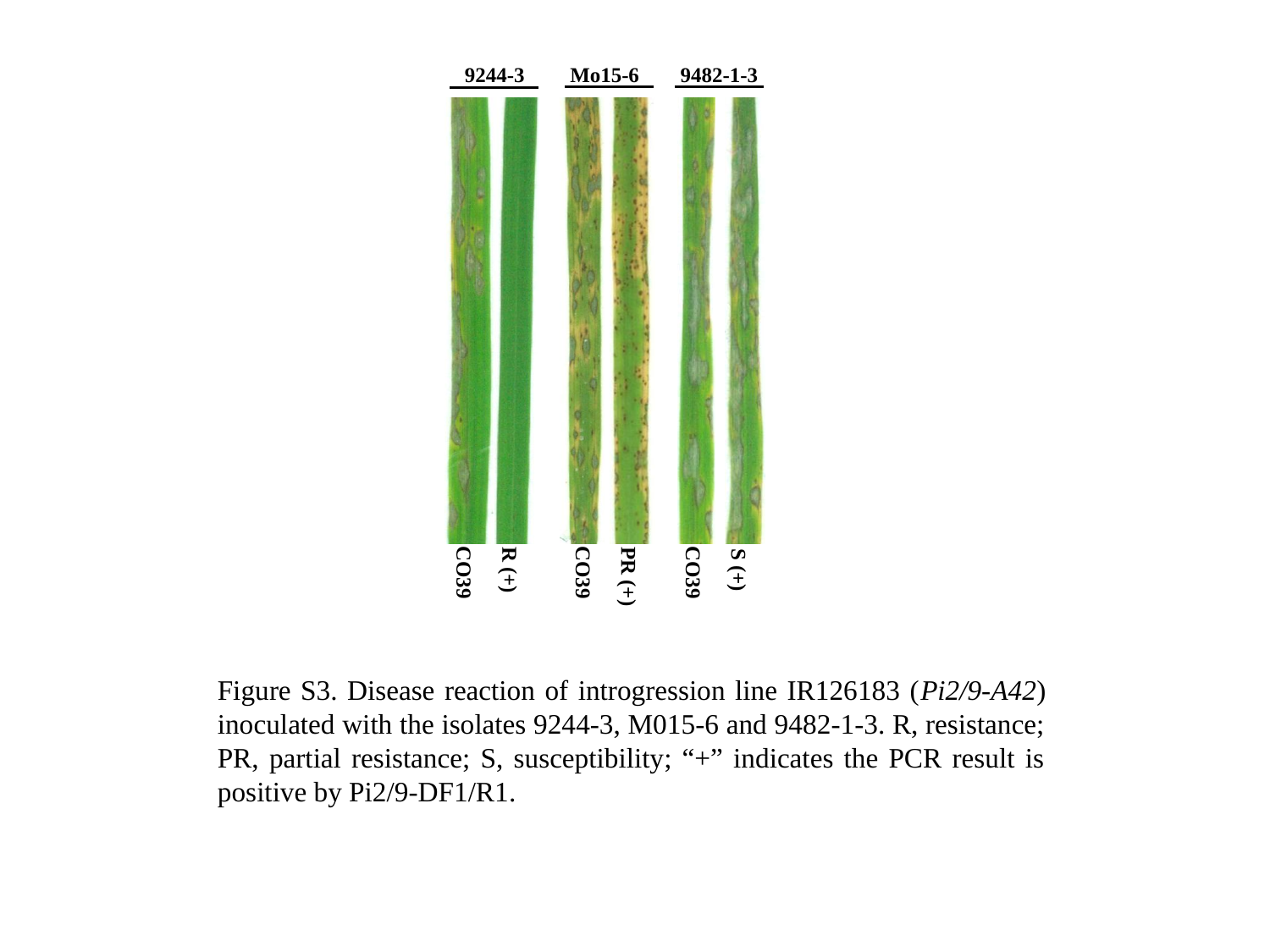

9244-3
Mo15-6
9482-1-3
R (+)
S (+)
PR (+)
CO39
CO39
CO39
Figure S3. Disease reaction of introgression line IR126183 (Pi2/9-A42) inoculated with the isolates 9244-3, M015-6 and 9482-1-3. R, resistance; PR, partial resistance; S, susceptibility; “+” indicates the PCR result is positive by Pi2/9-DF1/R1.

## Slide 5
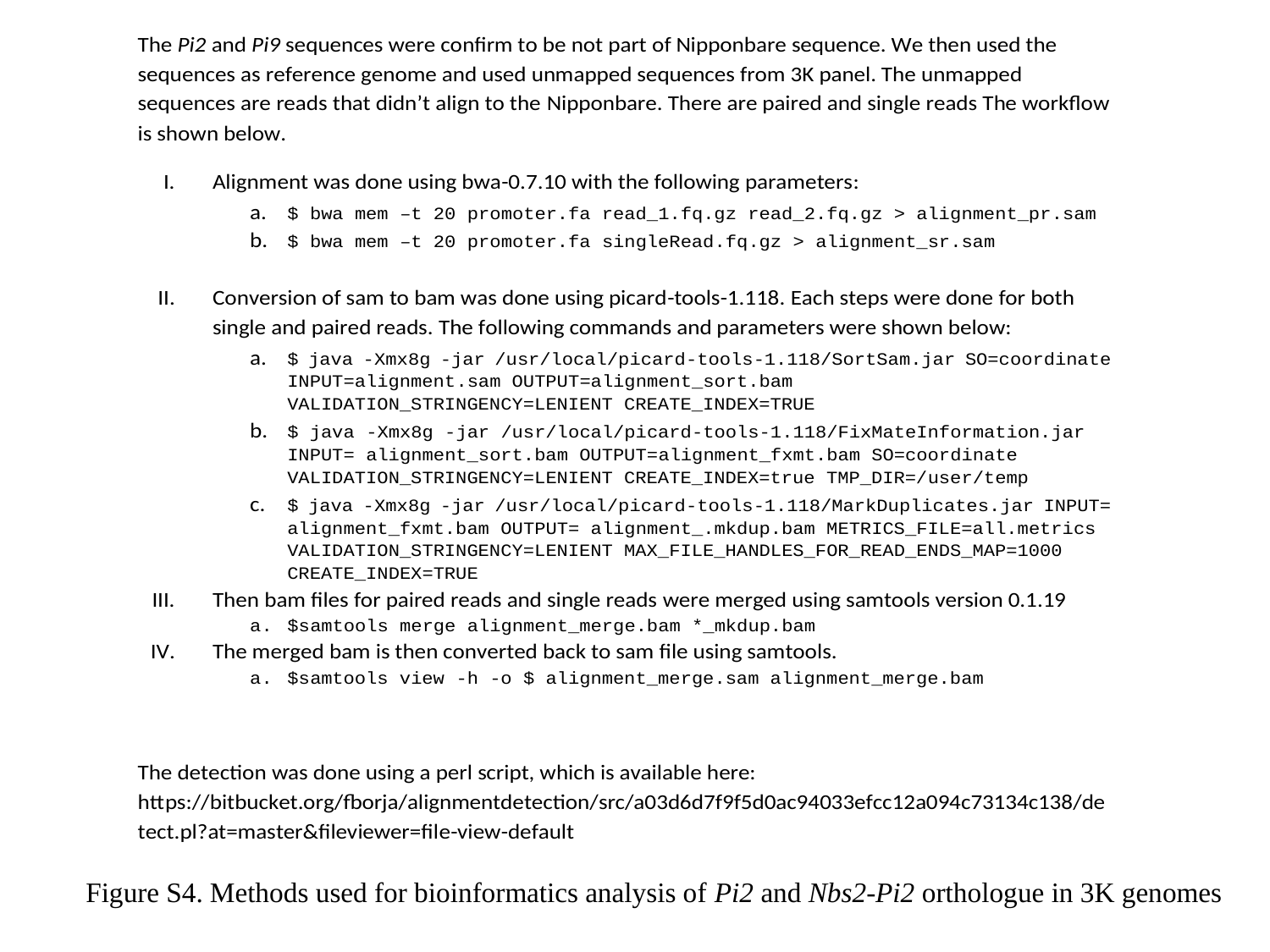

Figure S4. Methods used for bioinformatics analysis of Pi2 and Nbs2-Pi2 orthologue in 3K genomes
