# Supplementary material for: Identification of resistant germplasm containing novel resistance genes at or tightly linked to the Pi2/9 locus conferring broad-spectrum resistance against rice blast
Source: Rice (N Y). 2017 Aug 4;10:37. doi: 10.1186/s12284-017-0176-z (PMC5544663; doi:10.1186/s12284-017-0176-z)
Supplement: Supplementary file 2 — Selected rice panels and reaction of rice germplasm accessions against different isolate. Table S2. IRRI-bred blast-resistance lines (IRBLs) in the genetic background of Lijiangxintuanheigu (LTH). Table S3. Rice blast (M. oryzae) isolates used in this study and their collected place and year. (DOCX 83 kb) [file 12284_2017_176_MOESM2_ESM.docx]

**Title: Identification of resistant germplasm containing novel resistance genes at or tightly linked to the *Pi2/9* locus conferring broad-spectrum resistance against rice blast**

Gui Xiao^1,2^, Frances Nikki Borja^1^, Ramil Mauleon^1^, Jonas Padilla^1^, Mary Jeanie Telebanco-Yanoria^1^, Jianxia Yang^3^, Guodong Lu^3^, Maribel Dionisio-Sese^2^ & Bo Zhou^1*^

^1^Genetics and Biotechnology Division, International Rice Research Institute, DAPO Box 7777, Metro Manila, Philippines

^2^Institute of Biological Sciences, University of the Philippines Los Baños, Laguna 4031, Philippines

^3^Fujian Agriculture and Forest University, Fuzhou, 350002, China

**Table S1. Selected rice panels and reaction of rice germplasm accessions against different isolate**

| Panels | SEQ | IRGC Acc. No./ Entry/Designation | *M. oryzae* isolates^a^ | | | | | | | | | PCR amplification result^b^ |
| --- | --- | --- | --- | --- | --- | --- | --- | --- | --- | --- | --- | --- |
|  |  |  | 1 | 2 | 3 | 4 | 5 | 6 | 7 | 8 | 9 |  |
| **Panel Ι** | A1 | 121222 | R | R | R | R | R | R | R | R | R | – |
|  | A2 | 121233 | R | R | R | R | R | R | R | R | R | – |
|  | A3 | 121253 | R | R | R | R | R | R | R | R | R | – |
|  | A4 | 121266 | R | R | R | R | R | R | R | R | R | – |
|  | A5 | 121299 | R | R | R | R | R | R | R | R | R | + |
|  | A6 | 121302 | R | R | R | R | R | R | R | R | R | – |
|  | A7 | 121315 | R | R | R | R | R | R | S | S | R | + |
|  | A8 | 121323 | R | R | R | R | R | R | R | R | NA | – |
|  | A9 | 121330 | R | R | R | R | R | PR | R | R | R | – |
|  | A10 | 121383 | R | R | R | R | R | R | R | R | R | + |
|  | A11 | 121384 | R | R | R | R | R | R | R | PR | R | – |
|  | A12 | 121392 | R | R | R | R | NA | R | R | R | R | + |
|  | A13 | 121396 | R | R | R | R | NA | S | NA | PR | R | – |
|  | A14 | 121431 | R | R | R | R | R | R | R | R | R | – |
|  | A15 | 121434 | R | R | R | R | R | R | R | R | R | + |
|  | A16 | 121444 | R | R | R | R | R | R | R | R | R | +^c^ |
|  | A17 | 121478 | R | R | R | R | R | R | R | R | R | – |
|  | A18 | 121480 | R | R | R | R | NA | R | NA | R | R | – |
|  | A19 | 121490 | R | R | R | R | R | R | R | R | R | + |
|  | A20 | 121500 | R | R | R | R | R | R | R | R | R | – |
|  | A21 | 121514 | R | R | R | R | NA | R | R | R | R | – |
|  | A22 | 121525 | R | R | R | R | R | R | R | R | R | – |
|  | A23 | 121536 | R | R | R | R | R | R | R | R | S | + |
|  | A24 | 121576 | R | R | R | R | R | R | R | R | R | – |
|  | A25 | 121607 | R | R | R | R | R | R | R | R | R | + |
|  | A26 | 121611 | R | R | R | R | R | PR | R | R | R | + |
|  | A27 | 121626 | R | R | R | R | R | R | R | R | R | – |
|  | A28 | 121632 | R | R | R | R | R | R | R | R | PR | + |
|  | A29 | 121635 | R | R | R | R | R | R | R | R | S | – |
|  | A30 | 121639 | R | R | R | R | R | R | R | R | R | – |
|  | A31 | 121660 | R | R | R | R | R | R | R | R | R | + |
|  | A32 | 121698 | R | R | R | R | R | R | R | R | PR | + |
|  | A33 | 121699 | R | R | R | R | R | R | R | R | PR | + |
|  | A34 | 121708 | R | R | R | R | R | PR | R | R | R | – |
|  | A35 | 121729 | R | R | R | R | R | R | R | R | R | – |
|  | A36 | 121730 | R | R | R | R | R | R | R | R | R | + |
|  | A37 | 121736 | R | R | R | R | R | R | R | R | PR | – |
|  | A38 | 121743 | R | R | R | R | R | R | R | R | R | – |
|  | A39 | 121744 | R | R | R | R | R | R | R | R | PR | + |
|  | A40 | 121749 | R | R | R | R | R | R | R | R | R | + |
|  | A41 | 121753 | R | R | R | R | R | R | R | R | R | + |
|  | A42 | 121755 | R | R | R | R | R | R | R | R | R | + |
|  | A43 | 121762 | R | R | R | R | R | R | R | R | PR | + |
|  | A44 | 121764 | R | R | R | R | R | R | R | R | R | + |
|  | A45 | 121765 | R | R | R | R | R | R | R | R | R | – |
|  | A46 | 121767 | R | R | R | R | R | R | R | R | R | – |
|  | A47 | 121804 | R | R | R | R | R | R | R | R | R | + |
|  | A48 | 121805 | R | R | R | R | R | R | R | R | R | + |
|  | A49 | 121843 | R | R | R | R | R | R | R | R | R | – |
|  | A50 | 121854 | R | R | R | R | R | R | R | R | R | – |
|  | A51 | 121856 | R | R | R | R | R | R | R | R | S | – |
|  | A52 | 121871 | R | R | R | R | R | R | R | R | S | – |
|  | A53 | 121884 | R | R | R | R | R | R | R | R | R | + |
|  | A54 | 121888 | R | R | R | R | R | R | R | R | R | + |
|  | A55 | 121898 | R | R | R | R | R | R | R | R | R | + |
| **Panel ΙI** | A56 | 121208 | PR | R | PR | S | Not tested | | | | | |
|  | A57 | 121210 | PR | R | S | PR |  |  |  |  |  |  |
|  | A58 | 121217 | PR | R | R | S |  |  |  |  |  |  |
|  | A59 | 121218 | PR | R | R | S |  |  |  |  |  |  |
|  | A60 | 121230 | R | R | R | S |  |  |  |  |  |  |
|  | A61 | 121240 | R | R | PR | S |  |  |  |  |  |  |
|  | A62 | 121250 | R | R | S | R |  |  |  |  |  |  |
|  | A63 | 121251 | R | R | R | S |  |  |  |  |  |  |
|  | A64 | 121256 | R | R | S | R |  |  |  |  |  |  |
|  | A65 | 121284 | PR | R | PR | S |  |  |  |  |  |  |
|  | A66 | 121291 | NA | R | S | PR |  |  |  |  |  |  |
|  | A67 | 121293 | PR | R | R | S |  |  |  |  |  |  |
|  | A68 | 121306 | PR | R | S | R |  |  |  |  |  |  |
|  | A69 | 121308 | PR | R | R | S |  |  |  |  |  |  |
|  | A70 | 121317 | PR | R | S | R |  |  |  |  |  |  |
|  | A71 | 121321 | PR | R | R | S |  |  |  |  |  |  |
|  | A72 | 121325 | PR | R | R | S |  |  |  |  |  |  |
|  | A73 | 121328 | PR | R | R | S |  |  |  |  |  |  |
|  | A74 | 121337 | PR | R | R | S |  |  |  |  |  |  |
|  | A75 | 121339 | PR | R | NA | S |  |  |  |  |  |  |
|  | A76 | 121344 | PR | R | R | S |  |  |  |  |  |  |
|  | A77 | 121348 | PR | R | R | S |  |  |  |  |  |  |
|  | A78 | 121359 | PR | R | R | S |  |  |  |  |  |  |
|  | A79 | 121365 | PR | R | R | S |  |  |  |  |  |  |
|  | A80 | 121366 | R | PR | S | R |  |  |  |  |  |  |
|  | A81 | 121371 | PR | R | R | S |  |  |  |  |  |  |
|  | A82 | 121389 | PR | R | R | S |  |  |  |  |  |  |
|  | A83 | 121391 | PR | R | R | S |  |  |  |  |  |  |
|  | A84 | 121401 | R | R | S | R |  |  |  |  |  |  |
|  | A85 | 121405 | PR | R | R | S |  |  |  |  |  |  |
|  | A86 | 121410 | PR | R | PR | S |  |  |  |  |  |  |
|  | A87 | 121411 | PR | R | R | S |  |  |  |  |  |  |
|  | A88 | 121423 | PR | R | PR | S |  |  |  |  |  |  |
|  | A89 | 121425 | PR | PR | PR | S |  |  |  |  |  |  |
|  | A90 | 121438 | PR | R | R | S |  |  |  |  |  |  |
|  | A91 | 121441 | PR | R | S | R |  |  |  |  |  |  |
|  | A92 | 121443 | PR | R | R | S |  |  |  |  |  |  |
|  | A93 | 121449 | PR | R | R | S |  |  |  |  |  |  |
|  | A94 | 121450 | R | R | S | R |  |  |  |  |  |  |
|  | A95 | 121457 | PR | R | R | S |  |  |  |  |  |  |
|  | A96 | 121460 | PR | R | S | PR |  |  |  |  |  |  |
|  | A97 | 121461 | PR | R | PR | S |  |  |  |  |  |  |
|  | A98 | 121463 | PR | R | PR | S |  |  |  |  |  |  |
|  | A99 | 121469 | R | R | PR | S |  |  |  |  |  |  |
|  | A100 | 121474 | PR | R | R | S |  |  |  |  |  |  |
|  | A101 | 121475 | R | NA | S | PR |  |  |  |  |  |  |
|  | A102 | 121483 | PR | R | R | S |  |  |  |  |  |  |
|  | A103 | 121491 | R | R | R | S |  |  |  |  |  |  |
|  | A104 | 121492 | PR | PR | R | S |  |  |  |  |  |  |
|  | A105 | 121498 | R | R | PR | S |  |  |  |  |  |  |
|  | A106 | 121504 | R | R | S | R |  |  |  |  |  |  |
|  | A107 | 121507 | R | R | R | S |  |  |  |  |  |  |
|  | A108 | 121510 | PR | PR | R | S |  |  |  |  |  |  |
|  | A109 | 121515 | PR | PR | PR | S |  |  |  |  |  |  |
|  | A110 | 121516 | PR | R | PR | S |  |  |  |  |  |  |
|  | A111 | 121517 | R | R | R | S |  |  |  |  |  |  |
|  | A112 | 121518 | PR | PR | PR | S |  |  |  |  |  |  |
|  | A113 | 121519 | R | R | R | S |  |  |  |  |  |  |
|  | A114 | 121526 | PR | R | R | S |  |  |  |  |  |  |
|  | A115 | 121529 | PR | R | R | S |  |  |  |  |  |  |
|  | A116 | 121532 | PR | R | R | S |  |  |  |  |  |  |
|  | A117 | 121534 | PR | R | R | S |  |  |  |  |  |  |
|  | A118 | 121537 | R | R | R | S |  |  |  |  |  |  |
|  | A119 | 121546 | R | R | S | PR |  |  |  |  |  |  |
|  | A120 | 121547 | R | R | S | PR |  |  |  |  |  |  |
|  | A121 | 121548 | PR | R | R | S |  |  |  |  |  |  |
|  | A122 | 121556 | PR | R | R | S |  |  |  |  |  |  |
|  | A123 | 121559 | R | R | R | S |  |  |  |  |  |  |
|  | A124 | 121567 | PR | PR | R | S |  |  |  |  |  |  |
|  | A125 | 121568 | PR | PR | PR | S |  |  |  |  |  |  |
|  | A126 | 121570 | R | PR | R | S |  |  |  |  |  |  |
|  | A127 | 121574 | PR | R | PR | S |  |  |  |  |  |  |
|  | A128 | 121575 | PR | R | R | S |  |  |  |  |  |  |
|  | A129 | 121589 | R | PR | S | R |  |  |  |  |  |  |
|  | A130 | 121590 | PR | R | R | S |  |  |  |  |  |  |
|  | A131 | 121600 | PR | R | R | S |  |  |  |  |  |  |
|  | A132 | 121606 | R | R | R | S |  |  |  |  |  |  |
|  | A133 | 121608 | R | PR | S | R |  |  |  |  |  |  |
|  | A134 | 121612 | PR | R | S | R |  |  |  |  |  |  |
|  | A135 | 121613 | PR | R | PR | S |  |  |  |  |  |  |
|  | A136 | 121625 | PR | R | R | S |  |  |  |  |  |  |
|  | A137 | 121636 | R | R | S | PR |  |  |  |  |  |  |
|  | A138 | 121637 | PR | R | PR | S |  |  |  |  |  |  |
|  | A139 | 121649 | R | R | S | R |  |  |  |  |  |  |
|  | A140 | 121653 | R | PR | S | R |  |  |  |  |  |  |
|  | A141 | 121655 | R | R | S | PR |  |  |  |  |  |  |
|  | A142 | 121656 | R | R | PR | S |  |  |  |  |  |  |
|  | A143 | 121662 | PR | R | PR | S |  |  |  |  |  |  |
|  | A144 | 121663 | R | R | S | R |  |  |  |  |  |  |
|  | A145 | 121665 | R | PR | PR | S |  |  |  |  |  |  |
|  | A146 | 121666 | PR | R | R | S |  |  |  |  |  |  |
|  | A147 | 121668 | PR | PR | S | PR |  |  |  |  |  |  |
|  | A148 | 121669 | R | PR | S | R |  |  |  |  |  |  |
|  | A149 | 121675 | PR | R | R | S |  |  |  |  |  |  |
|  | A150 | 121685 | PR | R | S | PR |  |  |  |  |  |  |
|  | A151 | 121690 | R | R | S | R |  |  |  |  |  |  |
|  | A152 | 121693 | R | R | R | S |  |  |  |  |  |  |
|  | A153 | 121703 | R | R | S | PR |  |  |  |  |  |  |
|  | A154 | 121705 | R | R | PR | S |  |  |  |  |  |  |
|  | A155 | 121722 | R | R | PR | S |  |  |  |  |  |  |
|  | A156 | 121728 | PR | R | S | R |  |  |  |  |  |  |
|  | A157 | 121734 | PR | PR | R | S |  |  |  |  |  |  |
|  | A158 | 121737 | PR | R | S | PR |  |  |  |  |  |  |
|  | A159 | 121740 | R | R | PR | S |  |  |  |  |  |  |
|  | A160 | 121746 | PR | R | S | PR |  |  |  |  |  |  |
|  | A161 | 121754 | R | R | PR | S |  |  |  |  |  |  |
|  | A162 | 121757 | R | R | S | R |  |  |  |  |  |  |
|  | A163 | 121761 | PR | R | R | S |  |  |  |  |  |  |
|  | A164 | 121763 | PR | R | S | PR |  |  |  |  |  |  |
|  | A165 | 121768 | PR | R | R | S |  |  |  |  |  |  |
|  | A166 | 121777 | PR | R | PR | S |  |  |  |  |  |  |
|  | A167 | 121778 | R | R | S | R |  |  |  |  |  |  |
|  | A168 | 121785 | PR | PR | R | S |  |  |  |  |  |  |
|  | A169 | 121793 | PR | R | S | R |  |  |  |  |  |  |
|  | A170 | 121794 | PR | R | R | S |  |  |  |  |  |  |
|  | A171 | 121801 | PR | R | S | R |  |  |  |  |  |  |
|  | A172 | 121802 | PR | R | R | S |  |  |  |  |  |  |
|  | A173 | 121808 | PR | R | R | S |  |  |  |  |  |  |
|  | A174 | 121809 | R | R | R | S |  |  |  |  |  |  |
|  | A175 | 121828 | PR | R | R | S |  |  |  |  |  |  |
|  | A176 | 121829 | R | R | PR | S |  |  |  |  |  |  |
|  | A177 | 121833 | R | R | S | PR |  |  |  |  |  |  |
|  | A178 | 121838 | PR | R | R | S |  |  |  |  |  |  |
|  | A179 | 121844 | PR | R | R | S |  |  |  |  |  |  |
|  | A180 | 121845 | PR | R | R | S |  |  |  |  |  |  |
|  | A181 | 121847 | R | R | S | R |  |  |  |  |  |  |
|  | A182 | 121848 | PR | R | S | R |  |  |  |  |  |  |
|  | A183 | 121849 | PR | R | R | S |  |  |  |  |  |  |
|  | A184 | 121850 | PR | R | S | R |  |  |  |  |  |  |
|  | A185 | 121853 | PR | R | R | S |  |  |  |  |  |  |
|  | A186 | 121857 | R | R | S | R |  |  |  |  |  |  |
|  | A187 | 121858 | PR | R | R | S |  |  |  |  |  |  |
|  | A188 | 121859 | R | NA | R | NA |  |  |  |  |  |  |
|  | A189 | 121861 | PR | PR | PR | S |  |  |  |  |  |  |
|  | A190 | 121862 | R | PR | PR | S |  |  |  |  |  |  |
|  | A191 | 121864 | R | NA | R | NA |  |  |  |  |  |  |
|  | A192 | 121869 | PR | R | PR | S |  |  |  |  |  |  |
|  | A193 | 121872 | R | R | R | S |  |  |  |  |  |  |
|  | A194 | 121873 | PR | R | PR | S |  |  |  |  |  |  |
|  | A195 | 121876 | NA | R | NA | R |  |  |  |  |  |  |
|  | A196 | 121878 | NA | PR | R | S |  |  |  |  |  |  |
|  | A197 | 121892 | PR | R | R | S |  |  |  |  |  |  |
|  | A198 | 121894 | PR | R | R | S |  |  |  |  |  |  |
|  | A199 | 121907 | NA | R | PR | NA |  |  |  |  |  |  |
|  | A200 | 121908 | PR | R | R | S |  |  |  |  |  |  |
| **Panel III** | A201 | 117265 | R | PR | **Not tested** | | | | | | | – |
|  | A202 | 117266 | R | R |  |  |  |  |  |  |  | – |
|  | A203 | 117268 | PR | R |  |  |  |  |  |  |  | – |
|  | A204 | 117271 | PR | R |  |  |  |  |  |  |  | – |
|  | A205 | 117272 | PR | R |  |  |  |  |  |  |  | – |
|  | A206 | 117277 | PR | R |  |  |  |  |  |  |  | – |
|  | A207 | 117282 | R | PR |  |  |  |  |  |  |  | – |
|  | A208 | 117434 | PR | R |  |  |  |  |  |  |  | + |
|  | A209 | 117444 | R | R |  |  |  |  |  |  |  | – |
|  | A210 | 117446 | PR | R |  |  |  |  |  |  |  | + |
|  | A211 | 117456 | R | R |  |  |  |  |  |  |  | – |
|  | A212 | 117460 | NA | PR |  |  |  |  |  |  |  | – |
|  | A213 | 117467 | R | PR |  |  |  |  |  |  |  | – |
|  | A214 | 117498 | PR | R |  |  |  |  |  |  |  | – |
|  | A215 | 117500 | R | PR |  |  |  |  |  |  |  | – |
|  | A216 | 117505 | R | PR |  |  |  |  |  |  |  | – |
|  | A217 | 117510 | R | R |  |  |  |  |  |  |  | – |
|  | A218 | 117522 | R | PR |  |  |  |  |  |  |  | – |
|  | A219 | 117529 | R | NA |  |  |  |  |  |  |  | – |
|  | A220 | 117539 | PR | R |  |  |  |  |  |  |  | – |
|  | A221 | 117551 | R | PR |  |  |  |  |  |  |  | – |
|  | A222 | 117560 | R | PR |  |  |  |  |  |  |  | – |
|  | A223 | 117563 | R | R |  |  |  |  |  |  |  | + |
|  | A224 | 117564 | R | R |  |  |  |  |  |  |  | + |
|  | A225 | 117571 | PR | R |  |  |  |  |  |  |  | – |
|  | A226 | 117586 | R | PR |  |  |  |  |  |  |  | + |
|  | A227 | 117589 | R | R |  |  |  |  |  |  |  | – |
|  | A228 | 117596 | R | R |  |  |  |  |  |  |  | – |
|  | A229 | 117602 | R | R |  |  |  |  |  |  |  | – |
|  | A230 | 117610 | R | NA |  |  |  |  |  |  |  | – |
|  | A231 | 117612 | R | R |  |  |  |  |  |  |  | + |
|  | A232 | 117615 | PR | R |  |  |  |  |  |  |  | – |
|  | A233 | 117621 | R | R |  |  |  |  |  |  |  | – |
|  | A234 | 117622 | PR | PR |  |  |  |  |  |  |  | – |
|  | A235 | 117628 | R | R |  |  |  |  |  |  |  | – |
|  | A236 | 117641 | R | PR |  |  |  |  |  |  |  | – |
|  | A237 | 117652 | R | R |  |  |  |  |  |  |  | – |
|  | A238 | 117658 | R | PR |  |  |  |  |  |  |  | – |
|  | A239 | 117671 | PR | NA |  |  |  |  |  |  |  | – |
|  | A240 | 117672 | R | R |  |  |  |  |  |  |  | – |
|  | A241 | 117673 | R | R |  |  |  |  |  |  |  | – |
|  | A242 | 117679 | R | R |  |  |  |  |  |  |  | + |
|  | A243 | 117694 | PR | NA |  |  |  |  |  |  |  | – |
|  | A244 | 117695 | PR | NA |  |  |  |  |  |  |  | + |
|  | A245 | 117698 | PR | PR |  |  |  |  |  |  |  | – |
|  | A246 | 117699 | R | R |  |  |  |  |  |  |  | – |
|  | A247 | 117701 | R | PR |  |  |  |  |  |  |  | – |
|  | A248 | 117702 | PR | NA |  |  |  |  |  |  |  | – |
|  | A249 | 117703 | PR | R |  |  |  |  |  |  |  | – |
|  | A250 | 117717 | R | PR |  |  |  |  |  |  |  | – |
|  | A251 | 117720 | R | R |  |  |  |  |  |  |  | – |
|  | A252 | 117721 | R | R |  |  |  |  |  |  |  | – |
|  | A253 | 117724 | R | NA |  |  |  |  |  |  |  | – |
|  | A254 | 117725 | PR | NA |  |  |  |  |  |  |  | – |
|  | A255 | 117726 | R | PR |  |  |  |  |  |  |  | – |
|  | A256 | 117732 | PR | NA |  |  |  |  |  |  |  | – |
|  | A257 | 117735 | R | NA |  |  |  |  |  |  |  | – |
|  | A258 | 117740 | R | NA |  |  |  |  |  |  |  | – |
|  | A259 | 117751 | R | R |  |  |  |  |  |  |  | + |
|  | A260 | 117755 | R | R |  |  |  |  |  |  |  | + |
|  | A261 | 117757 | PR | R |  |  |  |  |  |  |  | – |
|  | A262 | 117760 | R | R |  |  |  |  |  |  |  | + |
|  | A263 | 117761 | PR | R |  |  |  |  |  |  |  | + |
|  | A264 | 117762 | R | R |  |  |  |  |  |  |  | + |
|  | A265 | 117764 | R | PR |  |  |  |  |  |  |  | – |
|  | A266 | 117765 | R | PR |  |  |  |  |  |  |  | – |
|  | A267 | 117780 | PR | PR |  |  |  |  |  |  |  | – |
|  | A268 | 117783 | PR | PR |  |  |  |  |  |  |  | – |
|  | A269 | 117786 | PR | R |  |  |  |  |  |  |  | – |
|  | A270 | 117796 | PR | R |  |  |  |  |  |  |  | – |
|  | A271 | 117799 | R | R |  |  |  |  |  |  |  | – |
|  | A272 | 117800 | PR | R |  |  |  |  |  |  |  | – |
|  | A273 | 117801 | R | PR |  |  |  |  |  |  |  | – |
|  | A274 | 117802 | R | PR |  |  |  |  |  |  |  | – |
|  | A275 | 117803 | PR | R |  |  |  |  |  |  |  | – |
|  | A276 | 117805 | R | PR |  |  |  |  |  |  |  | – |
|  | A277 | 117814 | R | R |  |  |  |  |  |  |  | – |
|  | A278 | 117817 | PR | PR |  |  |  |  |  |  |  | – |
|  | A279 | 117821 | PR | R |  |  |  |  |  |  |  | – |
|  | A280 | 117822 | R | NA |  |  |  |  |  |  |  | – |
|  | A281 | 117824 | PR | PR |  |  |  |  |  |  |  | + |
|  | A282 | 117829 | R | R |  |  |  |  |  |  |  | + |
|  | A283 | 117831 | PR | PR |  |  |  |  |  |  |  | – |
|  | A284 | 117839 | PR | PR |  |  |  |  |  |  |  | + |
|  | A285 | 117843 | R | R |  |  |  |  |  |  |  | – |
|  | A286 | 117846 | PR | PR |  |  |  |  |  |  |  | + |
|  | A287 | 117847 | R | PR |  |  |  |  |  |  |  | – |
|  | A288 | 117848 | NA | PR |  |  |  |  |  |  |  | – |
|  | A289 | 117849 | PR | PR |  |  |  |  |  |  |  | – |
|  | A290 | 117850 | R | R |  |  |  |  |  |  |  | – |
|  | A291 | 117851 | PR | PR |  |  |  |  |  |  |  | – |
|  | A292 | 117857 | R | R |  |  |  |  |  |  |  | – |
|  | A293 | 117860 | PR | R |  |  |  |  |  |  |  | – |
|  | A294 | 117862 | PR | PR |  |  |  |  |  |  |  | – |
|  | A295 | 117864 | R | PR |  |  |  |  |  |  |  | + |
|  | A296 | 117869 | PR | PR |  |  |  |  |  |  |  | – |
|  | A297 | 117881 | PR | PR |  |  |  |  |  |  |  | + |
|  | A298 | 117885 | PR | NA |  |  |  |  |  |  |  | – |
|  | A299 | 117890 | PR | NA |  |  |  |  |  |  |  | – |
|  | A300 | 117891 | R | R |  |  |  |  |  |  |  | + |
|  | A301 | 117893 | PR | NA |  |  |  |  |  |  |  | – |
|  | A302 | 117906 | R | R |  |  |  |  |  |  |  | – |
|  | A303 | 117912 | PR | R |  |  |  |  |  |  |  | – |
|  | A304 | 117918 | R | R |  |  |  |  |  |  |  | – |
|  | A305 | 117920 | R | R |  |  |  |  |  |  |  | + |
|  | A306 | 117922 | R | NA |  |  |  |  |  |  |  | – |
|  | A307 | 117926 | R | NA |  |  |  |  |  |  |  | + |
|  | A308 | 117931 | R | PR |  |  |  |  |  |  |  | + |
|  | A309 | 117932 | R | PR |  |  |  |  |  |  |  | + |
|  | A310 | 117936 | R | PR |  |  |  |  |  |  |  | – |
|  | A311 | 117937 | R | NA |  |  |  |  |  |  |  | – |
|  | A312 | 117939 | NA | R |  |  |  |  |  |  |  | – |
|  | A313 | 117944 | R | R |  |  |  |  |  |  |  | – |
|  | A314 | 120853 | R | NA |  |  |  |  |  |  |  | – |
|  | A315 | 120858 | R | R |  |  |  |  |  |  |  | – |
|  | A316 | 120869 | R | NA |  |  |  |  |  |  |  | – |
|  | A317 | 120870 | PR | NA |  |  |  |  |  |  |  | – |
|  | A318 | 120871 | PR | PR |  |  |  |  |  |  |  | + |
|  | A319 | 120872 | R | PR |  |  |  |  |  |  |  | – |
|  | A320 | 120874 | R | R |  |  |  |  |  |  |  | – |
|  | A321 | 120875 | R | NA |  |  |  |  |  |  |  | – |
|  | A322 | 120878 | PR | PR |  |  |  |  |  |  |  | + |
|  | A323 | 120892 | PR | R |  |  |  |  |  |  |  | – |
|  | A324 | 120899 | PR | NA |  |  |  |  |  |  |  | – |
|  | A325 | 120905 | PR | R |  |  |  |  |  |  |  | + |
|  | A326 | 120906 | PR | PR |  |  |  |  |  |  |  | + |
|  | A327 | 120911 | PR | R |  |  |  |  |  |  |  | – |
|  | A328 | 120913 | PR | R |  |  |  |  |  |  |  | – |
|  | A329 | 120919 | R | R |  |  |  |  |  |  |  | – |
|  | A330 | 120939 | PR | PR |  |  |  |  |  |  |  | – |
|  | A331 | 120947 | PR | R |  |  |  |  |  |  |  | + |
|  | A332 | 120950 | R | R |  |  |  |  |  |  |  | + |
|  | A333 | 120951 | R | R |  |  |  |  |  |  |  | – |
|  | A334 | 120953 | R | R |  |  |  |  |  |  |  | – |
|  | A335 | 120958 | PR | R |  |  |  |  |  |  |  | + |
|  | A336 | 120976 | R | R |  |  |  |  |  |  |  | – |
|  | A337 | 121014 | R | PR |  |  |  |  |  |  |  | – |
|  | A338 | 121016 | R | R |  |  |  |  |  |  |  | – |
|  | A339 | 121023 | PR | R |  |  |  |  |  |  |  | – |
|  | A340 | 121046 | PR | PR |  |  |  |  |  |  |  | – |
|  | A341 | 121068 | R | PR |  |  |  |  |  |  |  | – |
|  | A342 | 121071 | R | R |  |  |  |  |  |  |  | – |
|  | A343 | 121076 | PR | R |  |  |  |  |  |  |  | + |
|  | A344 | 121088 | R | R |  |  |  |  |  |  |  | – |
|  | A345 | 121089 | PR | R |  |  |  |  |  |  |  | – |
|  | A346 | 121090 | PR | R |  |  |  |  |  |  |  | + |
|  | A347 | 121100 | R | R |  |  |  |  |  |  |  | – |
|  | A348 | 121106 | R | PR |  |  |  |  |  |  |  | – |
|  | A349 | 121120 | R | PR |  |  |  |  |  |  |  | – |
|  | A350 | 121124 | PR | R |  |  |  |  |  |  |  | + |
|  | A351 | 121131 | R | R |  |  |  |  |  |  |  | + |
|  | A352 | 121141 | PR | R |  |  |  |  |  |  |  | + |
|  | A353 | 121147 | PR | R |  |  |  |  |  |  |  | + |
|  | A354 | 121155 | NA | PR |  |  |  |  |  |  |  | – |
|  | A355 | 121156 | NA | R |  |  |  |  |  |  |  | + |
|  | A356 | 121157 | NA | R |  |  |  |  |  |  |  | – |
| **Panel IV** | A357 | 117273 | R | S | **Not tested** | | | | | | | – |
|  | A358 | 117512 | S | S |  |  |  |  |  |  |  | – |
|  | A359 | 117616 | NA | NA |  |  |  |  |  |  |  | – |
|  | A360 | 117643 | S | S |  |  |  |  |  |  |  | – |
|  | A361 | 117646 | R | S |  |  |  |  |  |  |  | – |
|  | A362 | 117647 | S | S |  |  |  |  |  |  |  | – |
|  | A363 | 117714 | S | S |  |  |  |  |  |  |  | – |
|  | A364 | 117743 | R | S |  |  |  |  |  |  |  | – |
|  | A365 | 117746 | S | S |  |  |  |  |  |  |  | – |
|  | A366 | 117748 | R | S |  |  |  |  |  |  |  | – |
|  | A367 | 117758 | S | S |  |  |  |  |  |  |  | – |
|  | A368 | 117759 | S | R |  |  |  |  |  |  |  | – |
|  | A369 | 117772 | NA | S |  |  |  |  |  |  |  | – |
|  | A370 | 117776 | S | S |  |  |  |  |  |  |  | – |
|  | A371 | 117778 | S | S |  |  |  |  |  |  |  | – |
|  | A372 | 117779 | S | S |  |  |  |  |  |  |  | – |
|  | A373 | 117794 | S | R |  |  |  |  |  |  |  | – |
|  | A374 | 120903 | S | S |  |  |  |  |  |  |  | – |
|  | A375 | 120908 | S | NA |  |  |  |  |  |  |  | – |
|  | A376 | 120915 | S | S |  |  |  |  |  |  |  | – |
|  | A377 | 120925 | S | NA |  |  |  |  |  |  |  | – |
|  | A378 | 120927 | S | S |  |  |  |  |  |  |  | – |
|  | A379 | 120962 | S | PR |  |  |  |  |  |  |  | + |
|  | A380 | 120977 | PR | S |  |  |  |  |  |  |  | – |
|  | A381 | 121000 | S | PR |  |  |  |  |  |  |  | – |
|  | A382 | 121009 | S | S |  |  |  |  |  |  |  | – |
|  | A383 | 121025 | S | S |  |  |  |  |  |  |  | – |
|  | A384 | 121033 | S | PR |  |  |  |  |  |  |  | – |
|  | A385 | 121243 | S | S |  |  |  |  |  |  |  | – |
|  | A386 | 121257 | S | S |  |  |  |  |  |  |  | – |
|  | A387 | 121369 | S | S |  |  |  |  |  |  |  | + |
|  | A388 | 121439 | S | S |  |  |  |  |  |  |  | – |
|  | A389 | 121481 | S | S |  |  |  |  |  |  |  | + |
|  | A390 | 121512 | PR | S |  |  |  |  |  |  |  | – |
|  | A391 | 121564 | S | PR |  |  |  |  |  |  |  | – |
|  | A392 | 121583 | S | R |  |  |  |  |  |  |  | – |
|  | A393 | 121585 | S | S |  |  |  |  |  |  |  | – |
|  | A394 | 121618 | PR | S |  |  |  |  |  |  |  | – |
|  | A395 | 121638 | S | R |  |  |  |  |  |  |  | – |
|  | A396 | 121652 | S | PR |  |  |  |  |  |  |  | – |
|  | A397 | 121666 | S | R |  |  |  |  |  |  |  | + |
|  | A398 | 121681 | R | S |  |  |  |  |  |  |  | – |
|  | A399 | 121697 | S | PR |  |  |  |  |  |  |  | – |
|  | A400 | 121739 | S | R |  |  |  |  |  |  |  | – |
|  | A401 | 121747 | PR | S |  |  |  |  |  |  |  | – |
|  | A402 | 121759 | S | R |  |  |  |  |  |  |  | – |
|  | A403 | 122281 | NA | NA |  |  |  |  |  |  |  | – |
|  | A404 | 122295 | NA | NA |  |  |  |  |  |  |  | – |
|  | A405 | 122307 | NA | NA |  |  |  |  |  |  |  | – |
|  | A406 | 122400 | NA | NA |  |  |  |  |  |  |  | – |
| **Panel V** | A407 | COLOMBIAXXI | **Not tested** | | | | | | | | | – |
|  | A408 | CYPRESS |  |  |  |  |  |  |  |  |  | – |
|  | A409 | FEDEARROZ50 |  |  |  |  |  |  |  |  |  | – |
|  | A410 | INIATACUARI |  |  |  |  |  |  |  |  |  | – |
|  | A411 | IR01W105 |  |  |  |  |  |  |  |  |  | – |
|  | A412 | IR05N412 |  |  |  |  |  |  |  |  |  | – |
|  | A413 | IR06M143 |  |  |  |  |  |  |  |  |  | – |
|  | A414 | IR09A228 |  |  |  |  |  |  |  |  |  | – |
|  | A415 | IR10A134 |  |  |  |  |  |  |  |  |  | – |
|  | A416 | IR20 |  |  |  |  |  |  |  |  |  | – |
|  | A417 | IR26 |  |  |  |  |  |  |  |  |  | – |
|  | A418 | IR42 |  |  |  |  |  |  |  |  |  | – |
|  | A419 | IR44 |  |  |  |  |  |  |  |  |  | – |
|  | A420 | IR58 |  |  |  |  |  |  |  |  |  | – |
|  | A421 | IR65 |  |  |  |  |  |  |  |  |  | – |
|  | A422 | IR66 |  |  |  |  |  |  |  |  |  | + |
|  | A423 | IR70 |  |  |  |  |  |  |  |  |  | – |
|  | A424 | IR72 |  |  |  |  |  |  |  |  |  | – |
|  | A425 | IR74 |  |  |  |  |  |  |  |  |  | – |
|  | A426 | IR75084-15-3 |  |  |  |  |  |  |  |  |  | – |
|  | A427 | IR77390-6-1 |  |  |  |  |  |  |  |  |  | – |
|  | A428 | IR79971-B-191-B-B |  |  |  |  |  |  |  |  |  | – |
|  | A429 | IR8 |  |  |  |  |  |  |  |  |  | – |
|  | A430 | IRBB66 |  |  |  |  |  |  |  |  |  | – |
|  | A431 | IRRI112 |  |  |  |  |  |  |  |  |  | – |
|  | A432 | IRRI113 |  |  |  |  |  |  |  |  |  | – |
|  | A433 | IRRI122 |  |  |  |  |  |  |  |  |  | – |
|  | A434 | IRRI136 |  |  |  |  |  |  |  |  |  | – |
|  | A435 | IRRI141 |  |  |  |  |  |  |  |  |  | – |
|  | A436 | IRRI147 |  |  |  |  |  |  |  |  |  | – |
|  | A437 | IRRI148 |  |  |  |  |  |  |  |  |  | – |
|  | A438 | IRRI155 |  |  |  |  |  |  |  |  |  | + |
|  | A439 | IRRI156 |  |  |  |  |  |  |  |  |  | – |
|  | A440 | IRRI158 |  |  |  |  |  |  |  |  |  | – |
|  | A441 | IRRI160 |  |  |  |  |  |  |  |  |  | – |
|  | A442 | IRRI164 |  |  |  |  |  |  |  |  |  | + |
|  | A443 | SABITRI |  |  |  |  |  |  |  |  |  | – |
|  | A444 | IR10A136 |  |  |  |  |  |  |  |  |  | – |
|  | A445 | IR11N202 |  |  |  |  |  |  |  |  |  | – |
|  | A446 | IR12N235 |  |  |  |  |  |  |  |  |  | – |
|  | A447 | IR11W109 |  |  |  |  |  |  |  |  |  | – |
|  | A448 | IR12N281 |  |  |  |  |  |  |  |  |  | – |
|  | A449 | IR12A202 |  |  |  |  |  |  |  |  |  | – |
|  | A450 | IR10N134 |  |  |  |  |  |  |  |  |  | – |
|  | A451 | IR09N251 |  |  |  |  |  |  |  |  |  | – |
|  | A452 | IR12N198 |  |  |  |  |  |  |  |  |  | – |
|  | A453 | IR12N225 |  |  |  |  |  |  |  |  |  | – |
|  | A454 | IR09N532 |  |  |  |  |  |  |  |  |  | – |
|  | A455 | IR04A115 |  |  |  |  |  |  |  |  |  | – |
|  | A456 | IR04A285 |  |  |  |  |  |  |  |  |  | – |

R, PR and S indicate resistance, partial resistance and susceptible. NA denotes not applicable due to missing data.

IRGC Acc. No. indicates the international rice genebank collection accession number.

^a^Rice blast isolates: 1, CA89; 2, M64-1-3-9-1; 3, JMB8401; 4, M101-1-2-9-1; 5, 9239-4; 6, M39-1-3-8-1; 7, IK81-25; 8, M36-1-3-10-1; 9, CA41.

^b^PCR amplification was conducted using the primer Pi2/9-DF1/DR1, “+” indicate the presence of the *Pi2-*orthologue, “–” indicate no *Pi2-*orthologue.

^c^A16 produced an amplicon with a size larger than an expected one.

**Table S2. IRRI-bred blast-resistance lines (IRBLs) in the genetic background of Lijiangxintuanheigu (LTH).**

| Entry No. | Designation | Target gene | Donors |
| --- | --- | --- | --- |
| IRBL 1 | IRBLa-A | *Pia* | AICHI ASAHI |
| IRBL 2 | IRBLa-C | *Pia* | CO 39 |
| IRBL 3 | IRBLi-F5 | *Pii* | FUJISAKA 5 |
| IRBL 4 | IRBLks-F5 | *Pik-s* | FUJISAKA 5 |
| IRBL 5 | IRBLks-S | *Pik-s* | SHIN 2 |
| IRBL 6 | IRBLk-ka | *Pik* | KANTO 51 |
| IRBL 7 | IRBLkp-K60 | *Pik-p* | K 60 |
| IRBL 8 | IRBLkh-K3 | *Pik-h* | K 3 |
| IRBL 9 | IRBLz-Fu | *Piz* | FUKUNISHIKI |
| IRBL 10 | IRBLz5-CA | *Piz5* | C101A51 |
| IRBL 11 | IRBLzt-T | *Piz-t* | TORIDE 1 |
| IRBL 12 | IRBLta-K1 | *Pita* | K1 |
| IRBL 13 | IRBLta-CT2 | *Pita* | C105TTP2L9 |
| IRBL 14 | IRBLb-B | *Pib* | BL 1 |
| IRBL 15 | IRBLt-K59 | *Pit* | K 59 |
| IRBL 16 | IRBLsh-S | *Pish* | SHIN 2 |
| IRBL 17 | IRBLsh-B | *Pish* | BL 1 |
| IRBL 18 | IRBL1-CL | *Pi1* | C101LAC |
| IRBL 19 | IRBL3-CP4 | *Pi3* | C104PKT |
| IRBL 20 | IRBL5-M | *Pi5(t)* | RIL 249 (Moro.) |
| IRBL 21 | IRBL7-M | *Pi7(t)* | RIL 29 (Moro.) |
| IRBL 22 | IRBL9-W | *Pi9* | WHD-1S-75-1-127 |
| IRBL 23 | IRBL12-M | *Pi12(t)* | RIL 10 |
| IRBL 24 | IRBL19-A | *Pi19* | AICHI ASAHI |
| IRBL 25 | IRBLkm-Ts | *Pik-m* | TSUYUAKE |
| IRBL 26 | IRBL20-IR24 | *Pi20* | ARL 24 |
| IRBL 27 | IRBLta2-Pi | *Pita2* | Pi No. 4 |
| IRBL 28 | IRBLta2-Re | *Pita2* | REIHO |
| IRBL 29 | IRBLta-CP1 | *Pita* | C101PKT |
| IRBL 30 | IRBL11-Zh | *Pi11(t)* | ZHAIYEQING |

**Table S3. Rice blast (*M. oryzae*) isolates used in this study and their collected place and year of collected.**

| Isolate | Place collected | Year collected | Isolate | Place collected | Year collected |
| --- | --- | --- | --- | --- | --- |
| BN111 | Los Baños, Laguna | 1990 | 9126-1 | Ubay, Bohol | 2014 |
| BN209 | unknown | unknown | 9244-3 | Ubay, Bohol | 2014 |
| Ca41 | Caliraya, Laguna | 1990 | 9406-3 | Ubay, Bohol | 2014 |
| Ca89 | Caliraya, Laguna | 1990 | 9475-1-3 | Ubay, Bohol | 2014 |
| IK81-25 | unknown | 1981 | 9482-1-3 | Ubay, Bohol | 2014 |
| JMB8401 | Caliraya, Laguna | 1985 | 9497-3 | Ubay, Bohol | 2014 |
| JMB840610 | Cuenca, Batangas | 1984 | IBN008 | Los Baños, Laguna | 2015 |
| M36-1-3-10-1 | Los Baños, Laguna | 1995 | IBN028 | Los Baños, Laguna | 2015 |
| M39-1-3-8-1 | Los Baños, Laguna | 1995 | MO15-1 | Ubay, Bohol | 2015 |
| M64-1-3-9-1 | Los Baños, Laguna | 1995 | MO15-6 | Ubay, Bohol | 2015 |
| M101-1-2-9-1 | Los Baños, Laguna | 1995 | MO15-19 | Ubay, Bohol | 2015 |
| PO6-6 | Los Baños, Laguna | 1980 | MO15-21 | Ubay, Bohol | 2015 |
| V86010 | Camarines Sur | 1986 | MO15-24 | Ubay, Bohol | 2015 |
| 9239-4 | Caliraya, Laguna | 1992 | MO15-27 | Ubay, Bohol | 2015 |
| 5008-3 | Ubay, Bohol | 2014 | MO15-110 | Ubay, Bohol | 2015 |
| 5092-3 | Ubay, Bohol | 2014 | MO15-125 | Ubay, Bohol | 2015 |
| 5167-1 | Ubay, Bohol | 2014 | MO15-226 | Ubay, Bohol | 2015 |
| 6161-1 | Ubay, Bohol | 2014 | MO15-244 | Ubay, Bohol | 2015 |
